# Dickkopf-1 promotes tumor progression of gefitinib- resistant non-small cell lung cancer through cancer cell-fibroblast interactions

**DOI:** 10.1186/s40164-025-00616-9

**Published:** 2025-03-01

**Authors:** Munkyung Choi, Yong June Choi, Young Joo Lee, Yujeong Lee, Jin-Haeng Chung, Keon Wook Kang

**Affiliations:** 1 College of Pharmacy, Research Institute of Pharmaceutical Sciences and Natural Products Research Institute, Seoul National University, Seoul, 08826 Republic of Korea; 2 Department of Pathology and Translational Medicine, Seoul National University Bundang Hospital, Seongnam, 13620 Republic of Korea

**Keywords:** Dickkopf-1, Cancer-associated fibroblast, Gefitinib-resistance, Intercellular interaction, Tumor microenvironment

## Abstract

**Background:**

Cancer cell-secreted proteins play a critical role in tumor progression and chemoresistance by influencing intercellular interactions within the tumor microenvironment. Investigating the intratumoral functions of these secretory proteins may provide insights into understanding and treating chemoresistant cancers. This study aims to identify potential anticancer target(s) in gefitinib-resistant non-small cell lung cancer (NSCLC), with a focus on secretory proteins and their effects on intercellular interactions.

**Methods:**

Differentially expressed secretory proteins were identified in gefitinib-resistant human NSCLC cell lines (PC9-GR and HCC827-GR), revealing an elevation in Dickkopf-1 (DKK1) expression and secretion. To elucidate the role of DKK1 in gefitinib-resistant cancer, the anticancer effects of a neutralizing antibody against DKK1 were evaluated in tumors comprising either cancer cells alone or cancer cells co-injected with human lung fibroblasts (MRC-5). Following the confirmation of the importance of cancer cell-fibroblast interactions in the protumorigenic activity of DKK1, the fibroblast traits modulated by DKK1 were further analyzed.

**Results:**

Gefitinib-resistant NSCLC cells exhibited increased DKK1 protein expression. Although elevated DKK1 levels were linked to poor prognosis, DKK1 did not directly affect cancer cell proliferation. However, DKK1 blockade showed significant anticancer effects in gefitinib-resistant tumors containing lung fibroblasts, suggesting that DKK1’s pro-tumorigenic roles are mediated through cancer cell-fibroblast interactions. DKK1 altered fibroblast characteristics, enhancing inflammatory fibroblast traits while diminishing myofibroblast traits in tumor microenvironment. These DKK1-induced changes were mediated via activation of the c-JUN pathway in fibroblasts. Moreover, DKK1 was identified as a potential anticancer target across various cancer types beyond gefitinib-resistant lung cancer.

**Conclusions:**

This study clarifies that DKK1 mediates interactions between cancer cells and fibroblasts in gefitinib-resistant lung cancer, contributing to tumor progression. Therefore, we propose DKK1 as a promising anticancer target for the treatment of gefitinib-resistant NSCLC.

**Supplementary Information:**

The online version contains supplementary material available at 10.1186/s40164-025-00616-9.

## Background

Cancer cells are known to actively secrete a variety of molecules, including growth factors, cytokines, and components of the extracellular matrix. These secreted molecules differ significantly from those of normal cells and have been documented across various cancer types [1]. The tumor microenvironment is composed of diverse cell types such as fibroblasts, endothelial cells, and immune cells, which interact to create a tumor-favorable environment [2]. Secreted proteins from cancer cells play critical roles in these intercellular interactions, influencing the properties and behavior of surrounding cells. For example, they can induce cancer stem cell transformation [3], activate fibroblasts to produce extracellular matrix and cytokines [4], and modify immune cells to adopt immune-suppressive phenotypes [5]. Thus, characterization of cancer cell-secreted proteins could be essential for understanding the tumor microenvironment and developing effective anticancer therapies.

Gefitinib, a tyrosine kinase inhibitor (TKI) targeting the epidermal growth factor receptor (EGFR), faces significant challenges due to the development of resistance. Third-generation TKIs, like osimertinib, have been developed to overcome resistance caused by the T790M mutation in *EGFR* gene [6]. Recent studies have also focused on overcoming EGFR-TKI resistance [7, 8]. These studies were based on the previous researches about resistant mechanisms to EGFR-TKIs. For example, co-targeting alternatively activated pathways, such as MET amplification, has demonstrated an enhanced anti-tumor effect of gefitinib [9]. However, despite these advancements, resistance to TKIs remains a significant challenge, necessitating further research particularly in cancer cells resistant to osimertinib or those lacking the T790M mutation. Investigating the changes in the characteristics of resistant cancer cells independent of EGFR signaling is crucial for this effort. Gefitinib-resistant cancer cells are known to exhibit alterations in metabolic characteristics [10, 11], intracellular signaling pathways [12], and the tumor microenvironment [13]. Among them, the tumor microenvironment, in particular, supports cancer cell survival through the distribution of growth factors, extracellular matrix components, and intratumoral immune cells [13, 14]. It also plays a pivotal role in determining the efficacy of immunotherapy drugs commonly used in lung cancer treatment [15]. Hence, studying the characteristics of the tumor microenvironment in gefitinib-resistant cancer could provide valuable insights. Chemo-resistant cancers often exhibit a unique secretome. These secretory proteins can directly contribute to anticancer drug resistance by physically blocking drug access or altering cancer cell properties [16]. Indirectly, they may influence anticancer drug resistance by modifying the characteristics of various intratumoral cells [16]. Therefore, understanding the secreted proteins in gefitinib-resistant cancer can is critical for comprehending the tumor microenvironment and guiding therapeutic strategies.

Here, we propose Dickkopf-1 (DKK1) as a key secretory protein influencing the tumor microenvironment in gefitinib-resistant cancer. Initially identified as an inhibitor of β-catenin-dependent Wnt signaling, DKK1 inhibits this pathway by competitive binding to LRP5/6, a Wnt receptor [17]. Subsequent studies have revealed that DKK1 is involved in additional signaling pathways. DKK1 promotes Wnt binding to Frizzled, another Wnt receptor, thereby activating the c-JUN pathway. It can also activate the AKT pathway by binding to CKAP4, a DKK1 receptor [18]. Recent studies have shown that DKK1 expression is elevated in many cancer types and contributes to tumor growth [19]. DKK1 has been implicated in inducing cancer stem cell transformation [20, 21] and modulating the immune environment to favor tumor progression by directly suppressing the activity of immune cells such as T lymphocytes [22]. In this study, we hypothesized that the increased expression of DKK1 in gefitinib-resistant non-small cell lung cancer (NSCLC) cells contributes to tumor progression. We aimed to investigate the previously unknown effects of DKK1 on fibroblasts within tumors. Collectively, we propose DKK1 as a potential anticancer target for the treatment of gefitinib-resistant NSCLC.

## Methods

### Cell culture

Human NSCLC cell lines (PC9 and HCC827), human breast cancer cell line (T47D), human prostate cancer cell line (PC3), mouse breast cancer cell line (4T1) were cultured in RPMI-1640 medium with 10% fetal bovine serum and 1% penicillin/streptomycin. Human breast cancer cell line (MDA-MB-231) and human kidney epithelial cell line (HEK293T, Ampho-phoenix) were cultured in Dulbecco’s modified Eagle’s medium (DMEM)/high glucose with 10% fetal bovine serum and 1% penicillin/streptomycin. Gefitinib-resistant cell lines were established in the previous study [23]. To generate over-expressing cell lines, cells were transduced with lentivirus. Lentivirus particles were generated using HEK293T cells transformed with psPAX2 (a gift from Didier Trono, Addgene plasmid #12260; http://n2t.net/addgene:12260; RRID: Addgene_12260), pMD2.G (a gift from Didier Trono, Addgene plasmid #12259; http://n2t.net/addgene:12259; RRID: Addgene_12259) and pLenti-C-Myc-DDK-IRES-NEO (Origene, Rockville, MD, USA) cloned with ORF sequence. DKK1 ORF clone was provided from Korea Human Gene Bank, Medical Genomics Research center, KRIBB, Korea. To generate knock-down cell lines, cells were transduced with retrovirus. Retrovirus particles were generated using Ampho-phoenix cell transformed with pGFP-V-RS (Origene) cloned with shRNA sequence. After transduction, stable cells were selected with proper antibiotics.

### Antibodies

Antibodies targeting active β-catenin (8814 S), LaminA/C (2032 S), p-AKT (9271 S), p-JNK (9251 S), p-c-JUN (9261 S), mTOR (2983 S), p-mTOR (5536 S), p70s6K (9202 S), p-p70s6K (9234 S), p-GSK3β (9336 S) were purchased from Cell Signaling Technology (Danvers, MA, USA). Antibodies targeting β-catenin (sc-7963), AKT (sc-5298), c-JUN (sc-1694), GSK3β (sc-9166) were purchased from Santa Cruz Biotechnology (Dallas, TX, USA). Antibodies targeting DKK1 (10170-R015, SinoBiological, Beijing, China), glyceraldehyde 3-phosphate dehydrogenase (GAPDH, CB1001, Merck Millipore, Burlington, MA, USA), JNK (3496-1, Epitomics, Burlingame, CA, USA), Col1a1 (PB9938, Boster Biological Technology, Pleasanton, CA, USA), α-smooth muscle actin (α-SMA, A5228, Sigma-Aldrich, St. Louis, MO, USA) were used. Neutralization antibodies for animal experiments (IgG4 and DKN-01) were provided from Leap Therapeutics (Cambridge, MA, USA). CCL2 antibody used in immunohistochemistry was purchased from Sigma-Aldrich (MABN712, St. Louis, MO, USA).

### Western blot

For western blot analysis, whole cell or tissue lysates were obtained using cell lysis buffer (10 mM Tris, 100 mM NaCl, 1 mM EGTA, 10% Glycerol, 1% TritonX-100, and 30 mM sodium pyrophosphate). After boiling at 100℃ for 5 min, the prepared protein samples were used for sodium dodecyl sulfate-polyacrylamide gel electrophoresis (SDS-PAGE) [24]. After SDS-PAGE, the separated proteins were transferred to nitrocellulose membranes (GE healthcare, Chicago, IL, USA). The membranes were blocked with 5% skim milk in phosphate-buffered saline (PBS) and then incubated with primary antibodies at 4℃ overnight. Next day, the membranes were washed three times with PBS and incubated with secondary antibodies at room temperature for 1 h. Western Chemiluminescent HRP Substrate (Merk Millipore) was used to detect Chemiluminescence signal.

### Cytokine array

Cytokine array was performed using Proteome Profiler Human XL Cytokine Array Kit (ARY022B, R&D systems, Minneapolis, MN, USA). Cells were plated on 6-well plate for 24 h, and cell culture supernatant was collected for cytokine array.

### Cell proliferation and cell migration assays

To evaluate cell proliferation rate, cells were seeded on 96-well plate. Cell confluency was measured every 4 h with IncuCyte S3(Essen Bioscience, Ann Arbor, MI, USA). Migratory phenotype was assessed by transwell or wound healing assay. For transwell assay, 3,000 cells in serum free media were seeded on the top transwell, and culture media with 10% FBS was loaded in the bottom chamber. The migrated cell number was counted every 4 h using IncuCyte Zoom (Essen Bioscience). For wound healing assay, cells were seeded on 96-well plate with 100% confluency and scratched with 96-well WoundMaker (Essen Bioscience). The wound confluency was measured every 4 h using IncuCyte Zoom.

### Reporter gene assay

pGL4.10 plasmid (Origene) was cloned with − 1000 to + 150 upstream sequence of human *DKK1* gene. Cells were transfected with the reporter plasmid and pGFP-V-RS (transfection efficiency control). After 24 h, GFP intensity were detected by IncuCyte S3 (Essen Bioscience), and then cells were lysed for the further evaluation of luciferase reporter activity. Dual luciferase assay kit (Promega, Madison, WI, USA) was used for reporter assay.

### In vivo experiments

All animal experiment procedures were approved by the Institutional Animal Care and Use Committee of Seoul National University (Approval #: SNU-220907-5-2, SNU-231031-2, SNU-211025-2, SNU-241108-4). Balb/c or Balb/c nude mice were purchased from Raon Bio (Yongin, Korea), and maintained in specific pathogen-free condition in Animal Center for Pharmaceutical Research at Seoul National University. For flank tumor models, 5 × 10^6^ HCC827-GR in the presence or absence of 5 × 10^6^ MRC-5 and 1 × 10^6^ of 4T1-Lv con or 4T1-mDKK1 OE cells were diluted in Dulbecco’s-PBS (dPBS), and injected subcutaneously. Tumor volume were measured every three days and calculated with (width^2^ x height)/2 and the experiments were terminated when tumor volume reached to maximal size(2cm^3^). In case of DKN-01 treatment study, mice were injected with IgG4 or DKN-01(10 mg/kg, twice a week) intraperitoneally. For tail-vein injection model, 6 × 10^5^ 4T1-LV con or 4T1-mDKK1 OE cells were diluted in dPBS and injected intravenously. After 14 days from tumor injection, the number of tumor nodule in lung was measured.

### 3D-spheroid assay

To form 3D-spheroid, 5,000 cells were plated on the ultra-low attachment round-bottom 96-well plate and centrifuged for 300 RCF for 5 min. After the formation of 3D-spheroid, the spheroids were incubated in RPMI-1640 medium with 10% fetal bovine serum and 1% penicillin/streptomycin and the volumes were measured every day and calculated with (width^2^ x height)/2 for 6 or 7 days.

### RNA isolation, RNA sequencing and quantitative reverse transcriptase-polymerase chain reaction (qRT-PCR)

Cells were lysed with TRIZOL reagent (Thermo Fisher Scientific, Waltham, MA, USA) at room temperature for 10 min. Chloroform was added to cell lysate and mixed vigorously for 15 s. The mixture was incubated for 2 min to separate aquatic layer. To clarify layers, samples were centrifuged at 4℃, 12,000 RCF for 15 min. The separated aquatic layer containing total RNA was precipitated with isopropanol for 20 min. The precipitated total RNA pellets were washed with 75% ethanol and prepared with RNA-free diethyl pyrocarbonate (DEPC) treated water. Prepared total RNA was used for further experiments. For RNA sequencing, all processes were performed by Macrogen (Seoul, Korea). For each sample, a library was independently prepared using 1 µg of total RNA with the Illumina TruSeq Stranded mRNA Sample Prep Kit (Illumina, Inc., #20020595, San Diego, CA, USA). The process began with purifying poly-A containing mRNA molecules using poly‐T-attached magnetic beads. The purified mRNA was then fragmented into small pieces using divalent cations at elevated temperatures. The fragmented RNA was reverse transcribed into first strand cDNA using SuperScript II reverse transcriptase (Invitrogen, #18064014, Waltham, MA, USA) and random primers. Second strand cDNA synthesis followed, utilizing DNA Polymerase I, RNase H, and dUTP. The cDNA fragments underwent end repair, addition of a single ‘A’ base, and adapter ligation. The products were purified and amplified by PCR to produce the final cDNA library. Libraries were quantified using KAPA Library Quantification kits for Illumina Sequencing platforms, following the qPCR Quantification Protocol Guide (KAPA BIOSYSTEMS, #KK4854, Wilmington, MA, USA), and their quality was assessed using TapeStation D1000 ScreenTape (Agilent Technologies, #5067–5582, Santa Clara, CA, USA). The indexed libraries were then submitted to an Illumina NovaSeq (Illumina) for paired-end (2 × 100 bp) sequencing. GSEA analysis was performed using GSEA v.4.3.2 [25, 26]. Gene ontology analysis is performed using gProfiler [27]. For qRT-PCR, cDNA was prepared with Maxime RT PreMix kit (iNtRON, Seoul, Korea). qRT-PCR was performed using cDNA with iQ™ SYBR^®^ Green Supermix (Bio-Rad, Hercules, CA, USA).

### Immunocytochemistry

Cells were seeded on cover glass and fixed with 4% paraformaldehyde. The fixed cells were permeabilized with 0.1% Triton X-100 for 15 min and then blocked with 1% bovine serum albumin in dPBS for 1 h. The blocked samples were incubated with primary antibody overnight and washed for three times before the following incubation with fluorophore-conjugated secondary antibody. ProLong Gold Antifade Mountant (Life technologies, Carlsbad, CA, USA) was used for mounting samples on the slide gasses. The images were produced with confocal microscope Leica TCS SP8 MP (Leica Microsystems, Wetzlar, Germany).

### Public data analysis

GSE121634 data set was obtained from NCBI GEO database and analyzed [28, 29]. The expression profile of DKK1 protein in lung cancer patient was obtained from oncoDB [30]. Overall survival rate of DKK1 low- or high-patients were obtained from Kaplan-Meier Plotter [31, 32]. DKK1 protein expression and gene dependency data were analyzed using DepMap [33–37]. Graphic images are Created with BioRender.com.

### Statistical analysis

Unpaired Student’s t-test or one-way analysis of variance (ANOVA) followed by the Tukey’s test was used for statistical analysis using GraphPad PRISM software (GraphPad Software Inc., San Diego, CA, USA). P-value < 0.05 were considered statistically significant.

## Results

### Identification of DKK1 as a key secretory protein in gefitinib-resistant NSCLC cells

**Fig. 1:**
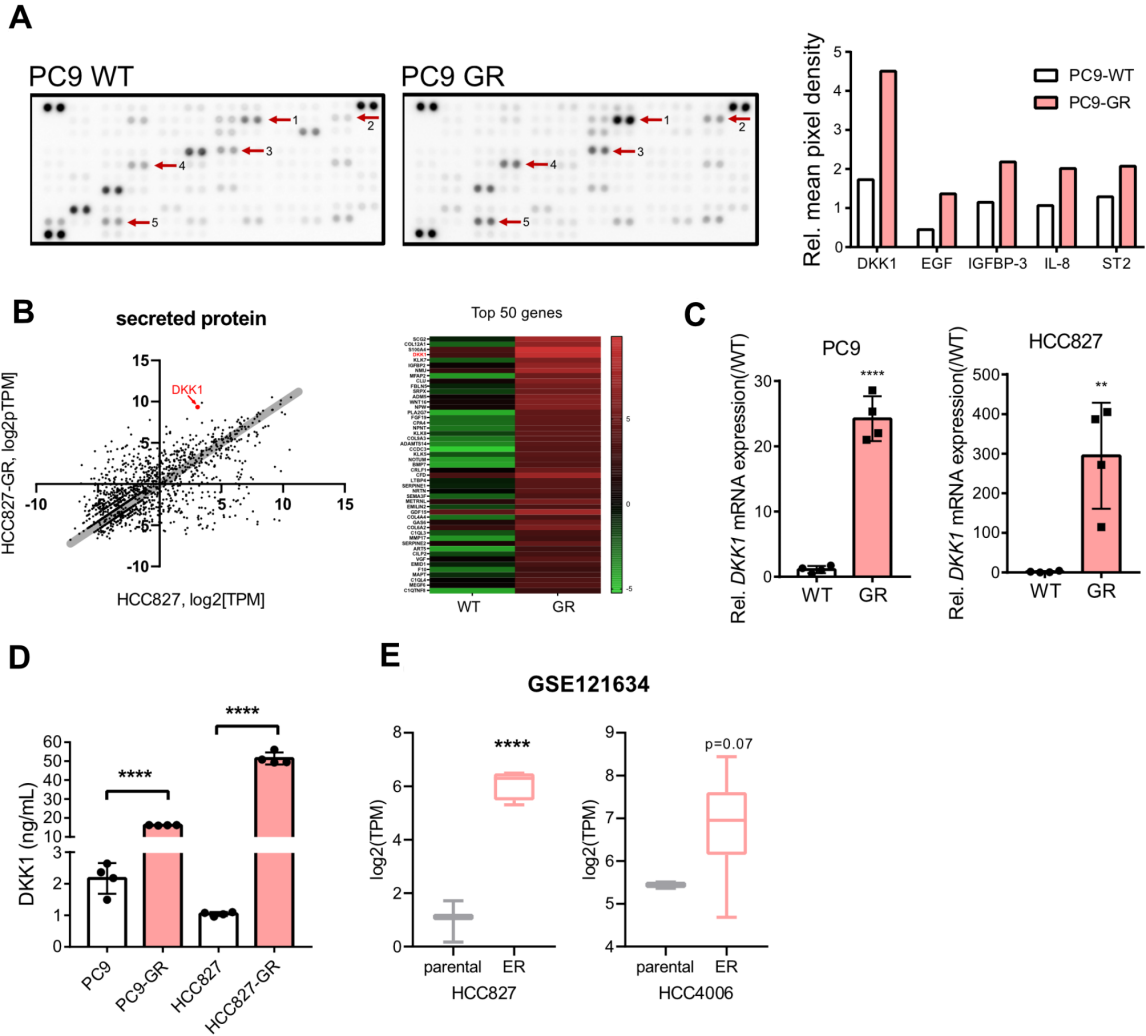
Enhanced DKK1 expression and secretion in gefitinib-resistant NSCLC cells (**A**) Cytokine array using PC9 and PC9-GR cell culture supernatant. (**B**) mRNA levels(left) and top 50 differentially expressed genes(right) among secreted protein genes between HCC827 and HCC827-GR. (**C** and **D**) *DKK1* mRNA (**C**) and protein levels (**D**) in PC9, PC9-GR, HCC827, and HCC827-GR. **(E)** DKK1 expression of HCC827 and HCC4006 compared with their erlotinib-resistant cells (ER). Data from GSE121634. All statistical significance of the differences was determined by unpaired two-tailed Student t-test. ns, non-significant; *, *P* < 0.05; **, *P* < 0.01; ***, *P* < 0.001; ****, *P* < 0.0001

Gefitinib-resistant (GR) variants of two NSCLC cell lines, PC9 (PC9-GR) and HCC827 (HCC827-GR), were previously established through prolonged gefitinib exposure [23] (supplementary Figure S1A). Given that chemoresistance can be shaped by the tumor microenvironment (TME) [38, 39], we hypothesized that the gefitinib-resistant cells (GR cells) might create a distinct TME compared to their parental cells. To investigate this, we focused on identifying differentially expressed secretory proteins in GR cells. A cytokine array analysis of PC9 and PC9-GR cells identified five candidate proteins (DKK1, EGF, IGFBP-3, IL-8, and ST2) that were highly upregulated in GR cells (Fig. 1A). Subsequent RNA sequencing of HCC827 and HCC827-GR cells revealed that among these proteins, only DKK1 was consistently overexpressed in both GR cell lines (Fig. 1B). This finding was confirmed by elevated DKK1 mRNA levels (Fig. 1C) and increased DKK1 protein secretion in the supernatant of both GR cell lines (Fig. 1D). In addition, analysis of public data (GSE121634) [28, 29] demonstrated increased DKK1 expression in lung cancer cells resistant to another EGFR-TKI, erlotinib, in HCC827 and HCC4006 cells. These data collectively suggest that GR cells consistently express and secrete high levels of DKK1 protein.

### DKK1 expression in GR cells is independent of acute EGFR signaling Inhibition

It has been reported that DKK1 expression is regulated by the β-catenin/TCF transcription complex, indicating a negative feedback loop in Wnt signaling and DKK1 expression [40]. Given the crosstalk between Wnt and EGFR signaling pathways [41], we hypothesized that DKK1 overexpression in GR cells might be a consequence of EGFR inhibition by gefitinib. To test whether DKK1 is induced by acute gefitinib exposure, PC9 and HCC827 cells were treated with gefitinib at concentrations that did not affect cell proliferation at 24 h (100 nM) or the concentration used for maintaining GR cells (1 µM). Neither DKK1 mRNA levels nor secreted DKK1 protein levels increased following gefitinib treatment in parental cells; rather, DKK1 secretion decreased (supplementary Figure S2A and S2B). These results can be explained by EGFR activity influencing post-translational regulation of β-catenin, where EGFR signaling inhibits GSK-3β, which in turn prevents β-catenin degradation [42]. Thus, the downregulation of EGFR signaling may result in the activation of GSK-3β and the subsequent degradation of β-catenin. Indeed, gefitinib treatment decreased phosphorylation of GSK-3β and reduced β-catenin protein levels in PC9 and HCC827 cells (supplementary Figure S2C).

### Adaptive phenotype of GR cells upregulates β-catenin transcriptional activity and DKK1 expression

**Fig. 2:**
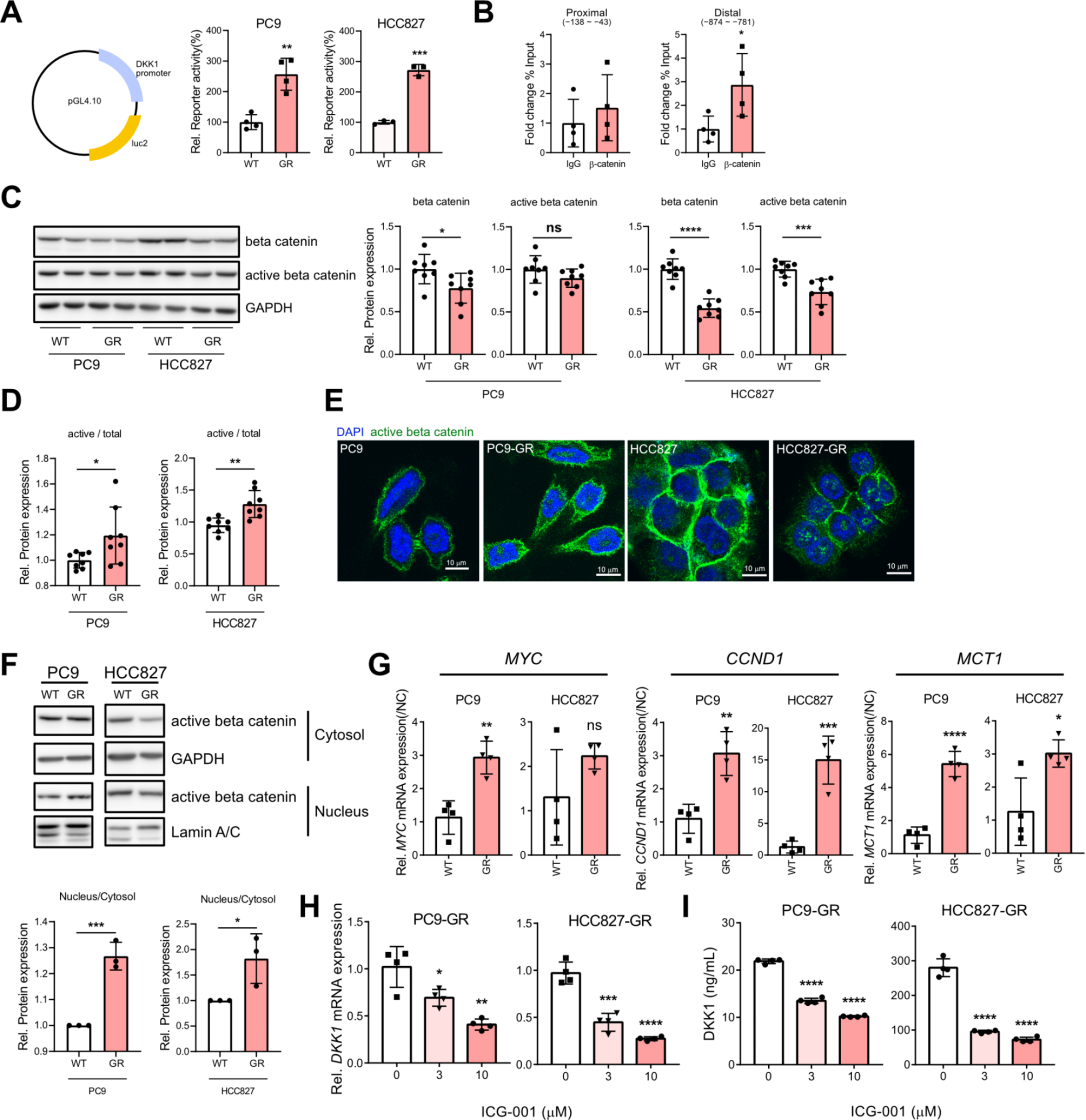
Enhanced transcriptional activity of β-catenin in gefitinib-resistant NSCLC cells. (**A**) Structure of DKK1 promoter reporter plasmid (left). The reporter activity of DKK1 promoter in gefitinib-resistant NSCLC cells(right). (**B**) Chromatin IP assay using non-phosphorylated(active) β-catenin antibody. Precipitated DNA was amplified by qPCR using primers target DKK1 promoter region containing TBE sites (proximal: −138 ~ − 43 / distal: −874 ~ − 781). Each points in bar graphs represent technical replicates. (**C**) Total and active β-catenin protein levels in PC9 and HCC827 parental and gefitinib-resistant cells. (**D**) Protein expression ratio of active/total β-catenin in gefitinib-resistant NSCLC cells. (**E**) Intracellular locations of active β-catenin (green) in PC9, PC9-GR, HCC827, and HCC827-GR. (**F**) Protein levels of active β-catenin in the cytosolic or nuclear fraction (top) and nucleus/cytosol expression ratio (bottom) of gefitinib-resistant NSCLC cells. (**G**) mRNA expression levels of β-catenin downstream genes. (**H** and **I**) mRNA expression level(**H**) and secreted protein level (**I**) of DKK1 in PC9-GR and HCC827-GR treated with ICG-001 for 24 h. All statistical significance of the differences was determined by unpaired two-tailed Student t-test. ns, non-significant; *, *P* < 0.05; **, *P* < 0.01; ***, *P* < 0.001; ****, *P* < 0.0001

Given that DKK1 overexpression in GR cells is not driven by EGFR inhibition, we hypothesized that it represents an adaptive response to prolonged gefitinib exposure. Since DKK1 transcription is activated by β-catenin, we tested whether β-catenin drives DKK1 transcription in GR cells. We constructed a luciferase reporter driven by the DKK1 promoter (-1000 to + 150 bp), which contains four TCF binding elements (TBE) to which the β-catenin/TCF transcription complex binds. The reporter exhibited increased activity in PC9-GR and HCC827-GR cells compared to their parental cells, indicating elevated β-catenin activity in GR cells (Fig. 2A). In addition, we performed Chromatin IP assay using non-phosphorylated(active) β-catenin antibody and primers target DKK1 promoter region containing TBE sites (proximal: −138 ~ − 43 / distal: −874 ~ − 781) [40]. As a result, distal region of DKK1 promoter was more precipitated by β-catenin antibody compared to IgG control indicating that β-catenin binds to DKK1 promoter, especially distal region containing TBEs (Fig. 2B). Since the protein stability of β-catenin is an important regulatory mechanism for β-catenin activity, we next attempted to determine whether the amount of β-catenin protein increased in GR cells. Although total β-catenin levels were reduced in GR cells (Fig. 2C), the proportion of active (dephosphorylated) β-catenin was higher (Fig. 2D). Immunofluorescence and immunoblotting after nuclear/cytosolic fractionation confirmed increased nuclear localization of active β-catenin in GR cells (Fig. 2E and F). Additionally, mRNA levels of several β-catenin target genes (*MYC*,* CCND1*,* MCT1*) were elevated in GR cells (Fig. 2G). Treatment with the β-catenin/TCF inhibitor ICG-001 reduced DKK1 mRNA and protein expression in GR cells (Fig. 2H and I), confirming that enhanced β-catenin transcriptional activity is responsible for high DKK1 expression in these cells.

### DKK1 blockade does not affect EGFR-TKI sensitivity and GR cell proliferation

Since GR cells express and secrete a high level of DKK1, we first tested whether DKK1 is involved in gefitinib sensitivity of GR cells. Despite the high levels of DKK1 in GR cells, it did not influence gefitinib resistance. Neither treatment with recombinant DKK1 protein nor manipulation of DKK1 expression altered gefitinib sensitivity in PC9 and HCC827 cells (supplementary Figure S3A-C). Thus, the enhanced expression of DKK1 itself may not be related to gefitinib resistance.

**Fig. 3:**
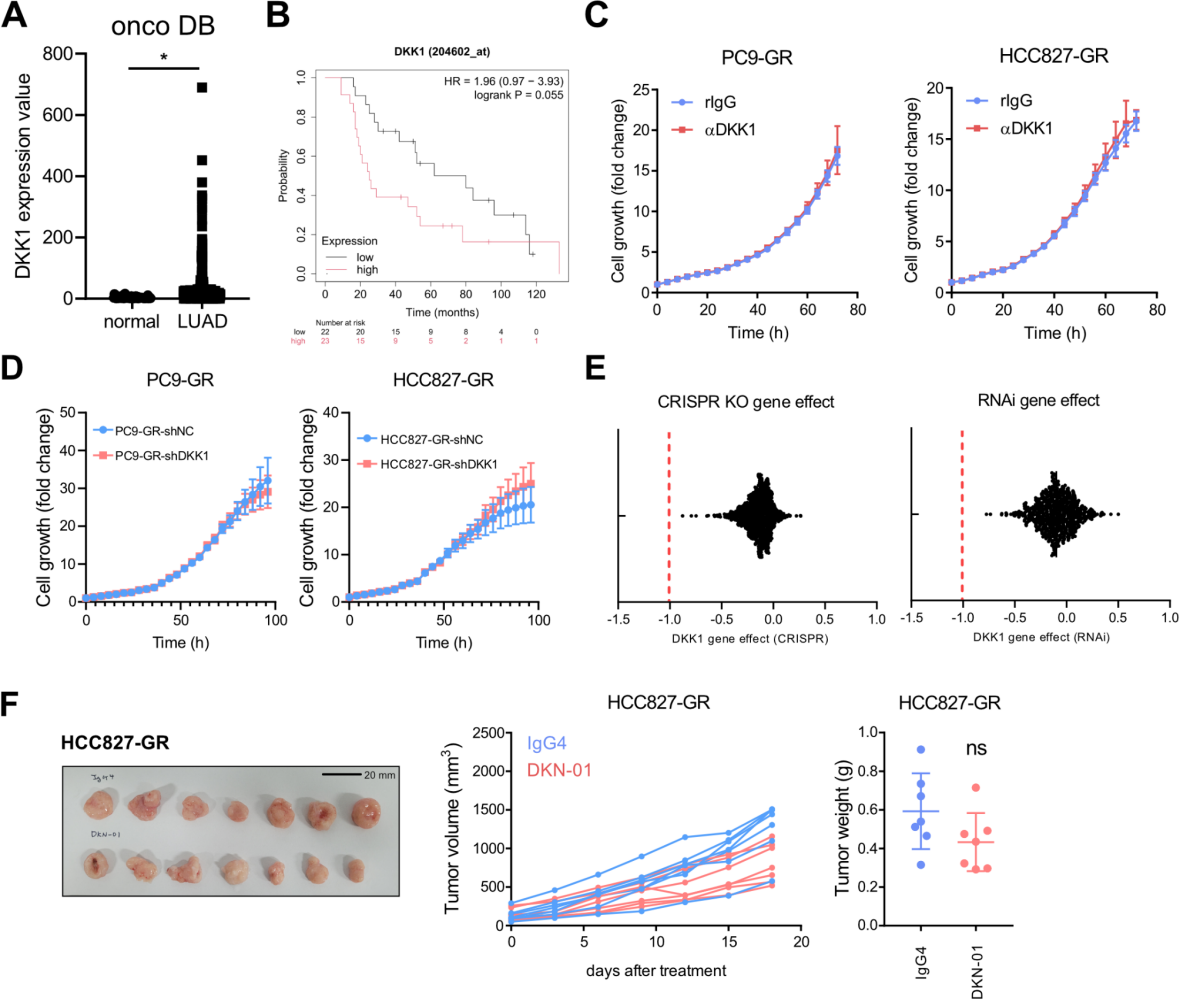
No effect of DKK1 blockade on proliferation and tumor growth of gefitinib-resistant NSCLC cells. (**A**) Expression values of DKK1 in healthy and lung adenocarcinoma (LUAD) patients. Data were collected from oncoDB. (**B**) The overall survival rate of DKK1-low or DKK1-high LUAD patients treated with chemotherapy and radiotherapy. Data were collected from Kaplan-Meier Plotter. (**C**) PC9-GR and HCC827-GR cell proliferation after treatment of 4 µg/mL DKK1 neutralization antibody. (**D**) Cell proliferation rate of PC9-GR and HCC827-GR expressing DKK1 shRNA. (**E**) DKK1 gene dependency of a variety of cancer cell lines. CRISPR knock-out and RNAi knock-down gene effect data were collected from Depmap. (**F**) Tumor volume and weight of HCC827-GR xenograft model (*n* = 7/group). Tumor-bearing mice were administered intraperitoneally with IgG4 or DKN-01 (10 mg/kg) twice a week. All statistical significance of the differences was determined by unpaired two-tailed Student t-test. ns, non-significant; *, *P* < 0.05; **, *P* < 0.01; ***, *P* < 0.001; ****, *P* < 0.0001

DKK1 has been reported to show increased expression in patients with various types of cancer and to be associated with malignancy of cancer [19]. Public data analysis using OncoDB [30] showed that mRNA expression of DKK1 was increased in LUAD patients (Fig. 3A). Furthermore, Kaplan-Meier Plotter analysis [31, 32] confirmed that the survival rate showed the negative correlation with DKK1 expression levels in patients treated with chemotherapy and radiotherapy (Fig. 3B). Therefore, we next tried to determine whether DKK1 promotes the cell proliferation of GR cells. DKK1 blockade with a neutralizing antibody did not affect GR cell proliferation (Fig. 3C), nor did DKK1 knockdown reduce GR cell growth (Fig. 3D). *DKK1* gene dependency analysis using Depmap database [33–37] also confirmed that neither CRISPR knockout nor RNA interference induced cell death in various cancer cell lines (Fig. 3E). Next, we tried to assess any changes in DKK1-mediated three downstream pathways in GR cells. Similarly, all of β-catenin pathway, c-JUN pathway, and CKAP4-AKT pathway were not altered by DKK1 knock-down or DKK1 neutralizing antibody in GR cells (supplementary Figure S4A and S4B). In agreement with these results, in vivo injection of DKN-01, a DKK1 neutralizing antibody, did not reduce tumor volume or weight in a subcutaneous xenograft model of HCC827-GR (Fig. 3F). Thus, DKK1 blockade does not directly affect GR cancer cell growth.

### DKK1’s role varies with tumor location

**Fig. 4:**
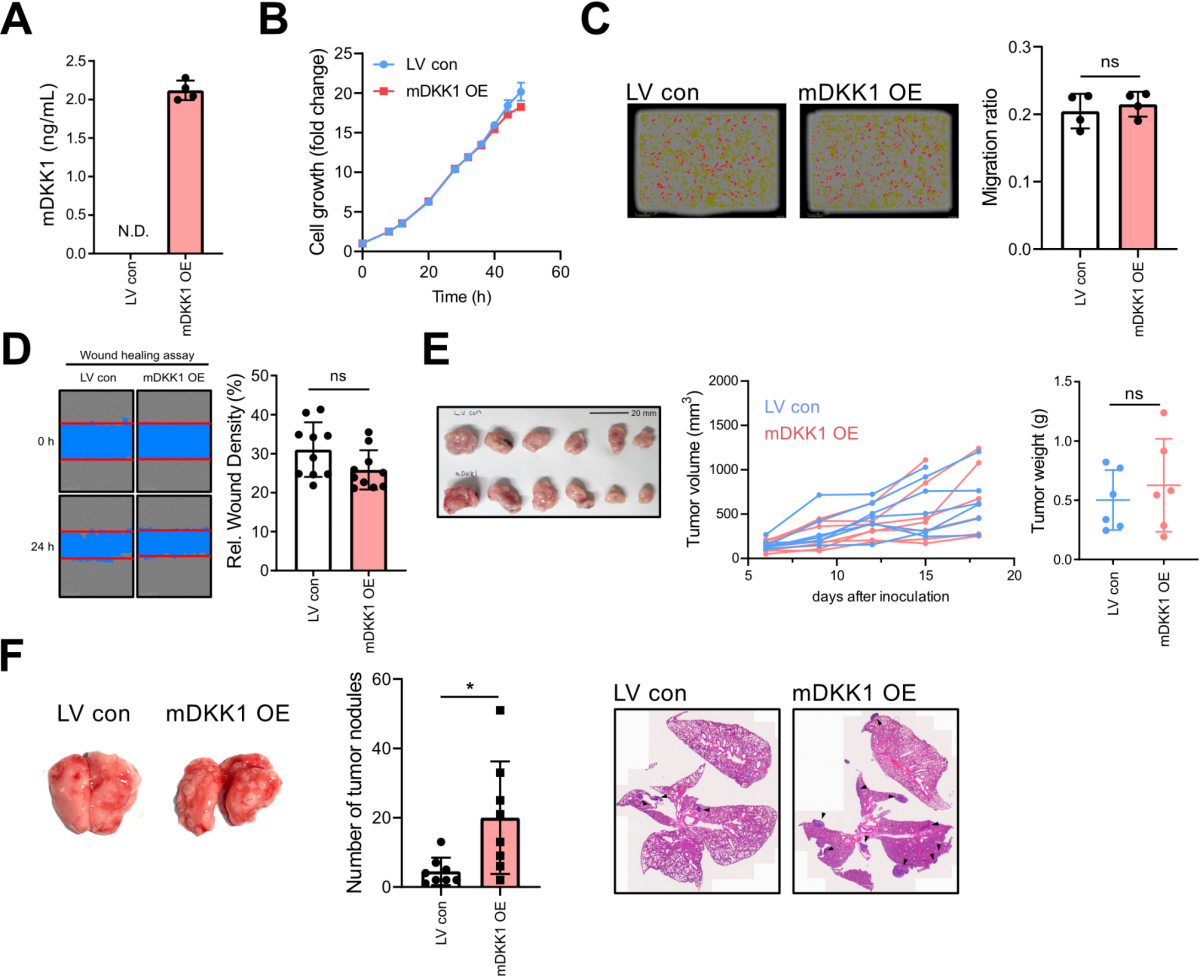
The influence of lung environment on the effect of DKK1 on tumor formation. (**A** and **B**) mDKK1 protein levels of cell supernatant(**A**) and cell proliferation rate(**B**) of 4T1-LV con and 4T1-mDKK1 OE. (**B**) Cell proliferation rate. (**C** and **D**) Cell migration was measured by transwell migration assay (**C**) and scratch wound healing assay (**D**). (**E**) Tumor volume and weight of subcutaneously implanted tumor with 4T1-LV con or 4T1-mDKK1 OE (*n* = 7/group). (**F**) Number of tumor nodules in the lungs following tail-vein injection of 4T1-LV con or 4T1-mDKK1 OE (left). Representative H&E staining images for each group (right)(*n* = 8/group). All statistical significance of the differences was determined by unpaired two-tailed Student t-test. ns, non-significant; *, *P* < 0.05; **, *P* < 0.01; ***, *P* < 0.001; ****, *P* < 0.0001

Although DKK1 blocking did not reduce subcutaneous tumor growth, patients with high DKK1 expression exhibit poorer survival (Fig. 3A and B). We hypothesized that this discrepancy might be due to differences in tumor location. To assess this, DKK1 overexpressing mouse cancer cells (4T1-mDKK1 OE) were generated using 4T1 mouse mammary cancer cell line, which has undetectable DKK1 secretion (Fig. 4A). Consistent with previous results, 4T1-mDKK1 OE did not show any changes in cell proliferation or migration ability compared to parental cells (Fig. 4B-D). However, in vivo, DKK1 overexpression significantly increased tumor engraftment in the lung, but did not affect tumor growth of the subcutaneously implanted cancer cells (Fig. 4E-F). This suggests that DKK1’s pro-tumor effects are dependent on the lung microenvironment.

### DKK1 blockade effectively reduces tumor growth in fibroblast-containing GR cells

Given that DKK1 is a secreted protein, we hypothesized GR cell-derived DKK1 influences other cell types within the TME. Among various cell types in TME, we focused on fibroblasts, which are abundant in the lung. Cancer-associated fibroblasts (CAFs) in the TME can be derived from variant types of cells including resident fibroblasts, endothelial cells, and mesenchymal stem cells. In NSCLC, CAFs can be originated from the resident lung fibroblasts [43].

**Fig. 5:**
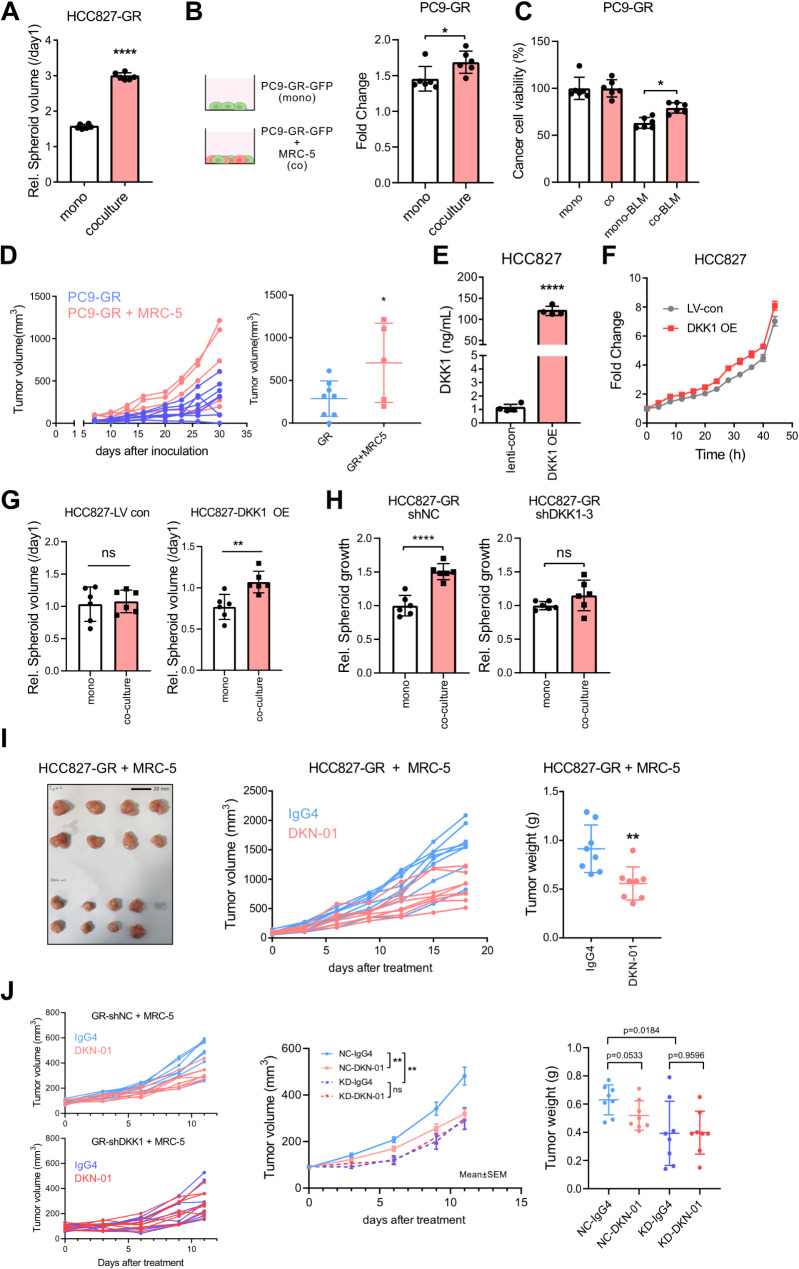
The effect of DKK1 blockade on fibroblast-containing tumor growth. (**A**) The fold change of volume of 3D spheroid containing HCC827-GR or HCC827-GR with MRC-5 after 7 days. (**B** and **C**) PC9-GR-GFP cell growth rates were measured by GFP-positive area after coculture with MRC-5 after 24 h. Growth rate of PC9-GR-GFP with or without MRC-5 were calculated by fold change of GFP-positive area to 0 h(**B**), and cell viability relative to vehicle group was measured by GFP-positive area after treatment of 5 µg/mL bleomycin for 24 h(**C**). (**D**) Tumor volume in a xenograft model of PC9-GR or PC9-GR + MRC-5 (*n* = 8 for PC9-GR, *n* = 5 for PC9-GR + MRC-5). (**E**) Secretion of DKK1 by HCC827-DKK1-OE and HCC827-LV-con. (**F**) Cell proliferation of HCC827-DKK1-OE compared with control cell. (**G**) HCC827-LV con (left) or HCC827-DKK1-OE (right) monocultured or cocultured with MRC-5 to form the 3D spheroids. The data shows the relative spheroid volumes after 6 days. (**H**) HCC827-GR-shNC (left) or HCC827-GR-shDKK1 (right) monocultured or cocultured with MRC-5 to form the 3D spheroids. The data shows the relative spheroid volumes after 7 days. (**I**) Tumor volume and weight in a flank xenograft model of HCC827-GR co-injected with MRC-5 (*n* = 8/group). Tumor-bearing mice were administered intraperitoneally with IgG4 or DKN-01 (10 mg/kg) twice a week. (**J**) Tumor volume and weight in a flank xenograft model of HCC827-GR-shNC + MRC-5 or HCC827-GR-shDKK1 (*n* = 8/group). Tumor-bearing mice were administered intraperitoneally with IgG4 or DKN-01 (10 mg/kg) twice a week. All statistical significance of the differences was determined by unpaired two-tailed Student t-test. ns, non-significant; *, *P* < 0.05; **, *P* < 0.01; ***, *P* < 0.001; ****, *P* < 0.0001

To assess whether lung fibroblasts promote GR tumor growth, we compared the growth of 3D spheroids composed of GR cells alone or GR cells co-cultured with the human lung fibroblast cell line, MRC-5. Co-culturing HCC827-GR with MRC-5 significantly increased spheroid growth (Fig. 5A). To examine the function of MRC-5 on cancer cell proliferation, GFP-expressing PC9-GR was co-cultured with MRC-5. Cell proliferation of PC9-GR detected by GFP area was elevated by MRC-5 co-culture (Fig. 5B). Moreover, bleomycin (5 µg/mL)-induced cytotoxicity was diminished by MRC-5 co-culture (Fig. 5C). In vivo co-injection of GR cells with MRC-5 resulted in larger tumors compared to GR cells alone (Fig. 5D). These results indicate that lung fibroblast can promote the growth of GR tumors. To investigate DKK1’s role in this interaction, we generated DKK1-overexpressing HCC827 cells (HCC827-DKK1 OE) (Fig. 5E). Consistent with previous data, HCC827-DKK1 OE showed a similar cell proliferation rate to parental cells (Fig. 5F). HCC827-LV con spheroid co-cultured with MRC-5 did not induce the changes of spheroid volume ratios relative to 1 day. However, in case of HCC827-DKK1 OE, spheroid volume ratios relative to 1 day were higher in MRC-5 co-cultured group (Fig. 5G). Conversely, DKK1 knockdown in HCC827-GR cells reduced the enhanced spheroid growth rate induced by MRC-5 co-culture (Fig. 5H). These results indicate the important role of DKK1 in facilitating cancer cell-fibroblast interactions and subsequent tumor growth.

Given that the minimal anti-tumor effect of DKN-01 in the HCC827-GR subcutaneous tumor model might be due to the absence of lung fibroblasts, we tested the antibody in an HCC827-GR/MRC-5 co-injection xenograft model. Treatment with DKN-01 significantly reduced tumor volume and weight compared to IgG4-treated control group (Fig. 5I). Additionally, we confirmed that the tumor reducing effect of DKN-01 is due to specific inhibition of DKK1, as DKN-01 did not decrease tumor volume of HCC827-GR-shDKK1 + MRC-5 tumors (Fig. 5J). These results indicate that DKK1 blockade is an effective anti-cancer strategy for gefitinib-resistant tumors, especially when fibroblasts are present in the TME.

### Cancer cell-derived DKK1 activates lung fibroblasts into inflammatory CAFs

**Fig. 6:**
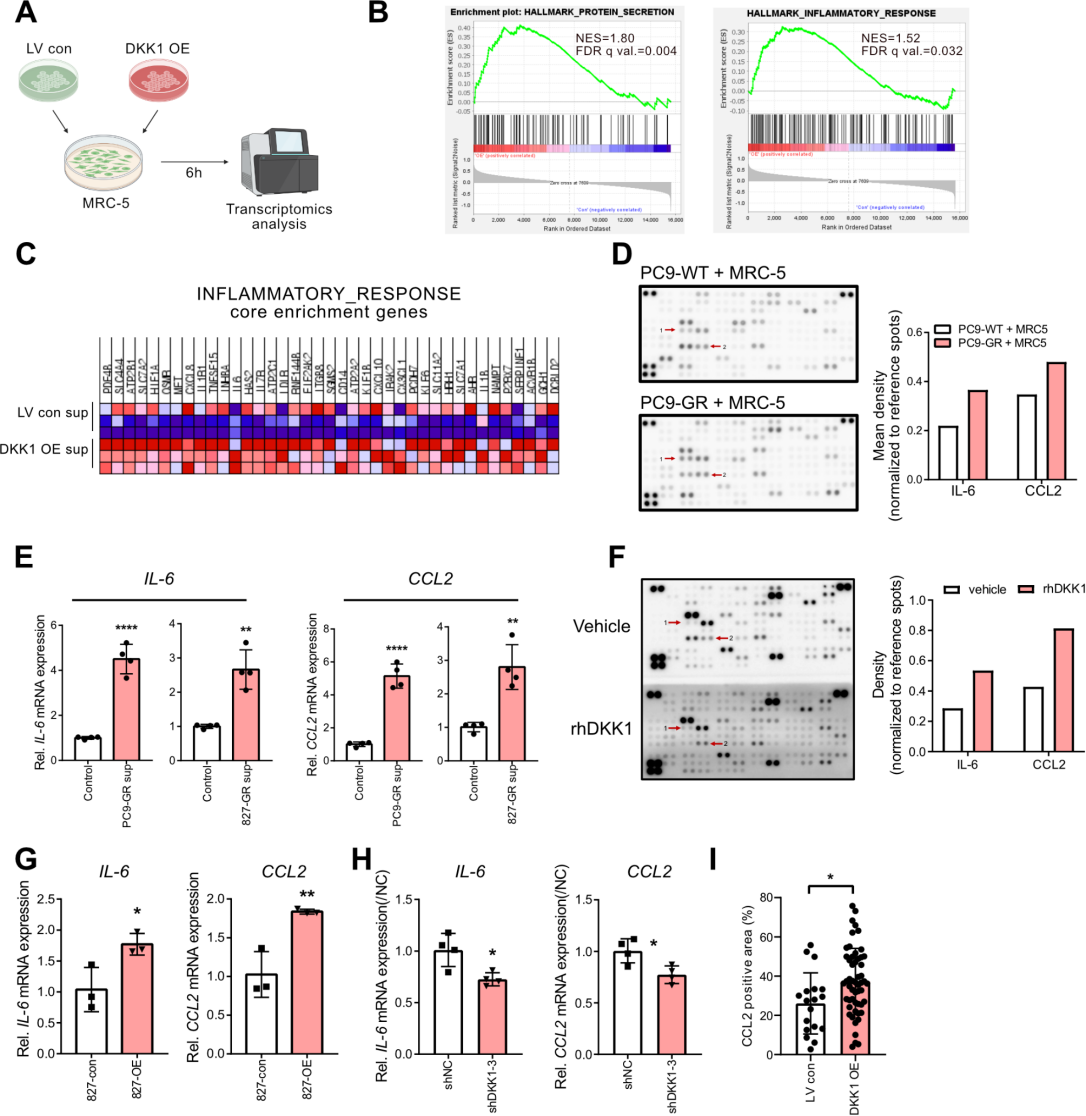
The effect of DKK1 on fibroblast activation. (**A**) Schematic figure of the transcriptomic analysis performed on MRC-5 cells treated with culture supernatants from HCC827-LV control or HCC827-DKK1-OE for 6 h. (**B**) Gene Set Enrichment Analysis (GSEA) of pathways upregulated in the DKK1 OE group. (**C**) Core enrichment genes from inflammatory response pathway in DKK1 OE group. (**D**) Cytokine array using PC9 or PC9-GR co-cultured with MRC-5 cell supernatant. (**E**) mRNA expression of *IL-6* and *CCL2* in MRC-5 treated with PC9-GR and HCC827-GR supernatant. (**F**) Cytokine array using MRC-5 supernatant treated with 200 ng/mL of recombinant DKK1 protein for 12 h. (**G** and **H**) *IL-6* and *CCL2* mRNA expression levels in MRC-5 treated with HCC827-DKK1-OE culture supernatant (**G**) or HCC827-GR-shDKK1 culture supernatant (**H**). (**I**) The analysis result of CCL2 positive area in tumor nodule section sample in Fig. 4F. All statistical significance of the differences was determined by unpaired two-tailed Student t-test. ns, non-significant; *, *P* < 0.05; **, *P* < 0.01; ***, *P* < 0.001; ****, *P* < 0.0001

We confirmed that cancer cell-derived DKK1 facilitates interactions between cancer cells and fibroblasts, thereby promoting tumor growth. To investigate whether DKK1 specifically drives lung fibroblast activation, MRC-5 cells were exposed to culture medium from HCC827-LV con or HCC827-DKK1 OE for 6 h, followed by RNA sequencing (Fig. 6A). Interestingly, GSEA analysis revealed that gene sets related to protein secretion and inflammatory response were enriched in the HCC827-DKK1 OE culture medium-treated group (Fig. 6B). Notably, several genes associated with the inflammatory response were upregulated in the DKK1 OE group (Fig. 6C). These findings led us to hypothesize that DKK1 can activate lung fibroblasts to mainly secrete cytokines.

CAFs are known to actively secrete various proteins. To examine changes in the secretory phenotype resulting from GR cell-mediated fibroblast activation, we performed a cytokine array using the supernatant from the direct co-culture of MRC-5 with PC9 or PC9-GR cells. The secretion of two inflammatory cytokines, IL-6 and CCL2, was notably increased in the PC9-GR co-culture (Fig. 6D). Supporting these findings, supernatants from PC9-GR and HCC827-GR cells induced mRNA expression of *IL-6* and *CCL2* in MRC5 cells (Fig. 6E).

To confirm that GR cell-mediated fibroblast activation is derived from high DKK1 expression, we examined fibroblast activation using recombinant human DKK1 protein. Similarly, DKK1 protein itself enhanced IL-6 and CCL2 secretion in MRC-5 cells (Fig. 6F). Additionally, culture medium from HCC827-DKK1 OE induced higher *IL-6* and *CCL2* expression than the control supernatant (Fig. 6G), while HCC827-GR-shDKK1 culture medium reduced *IL-6* and *CCL2* expression in MRC-5 cells (Fig. 6H). Furthermore, we analyzed CCL2 expression in the tumor nodule samples from Fig. 4F using immunohistochemistry and found that tumor nodules formed by DKK1 over-expressing cancer cells exhibited a higher CCL2-positive area (Fig. 6I). Since IL-6 and CCL2 are representative markers of inflammatory fibroblasts, our results indicate that cancer cell-derived DKK1 induces lung fibroblasts to adopt an inflammatory phenotype.

### DKK1 diminishes myofibroblast characteristics of lung fibroblasts

**Fig. 7:**
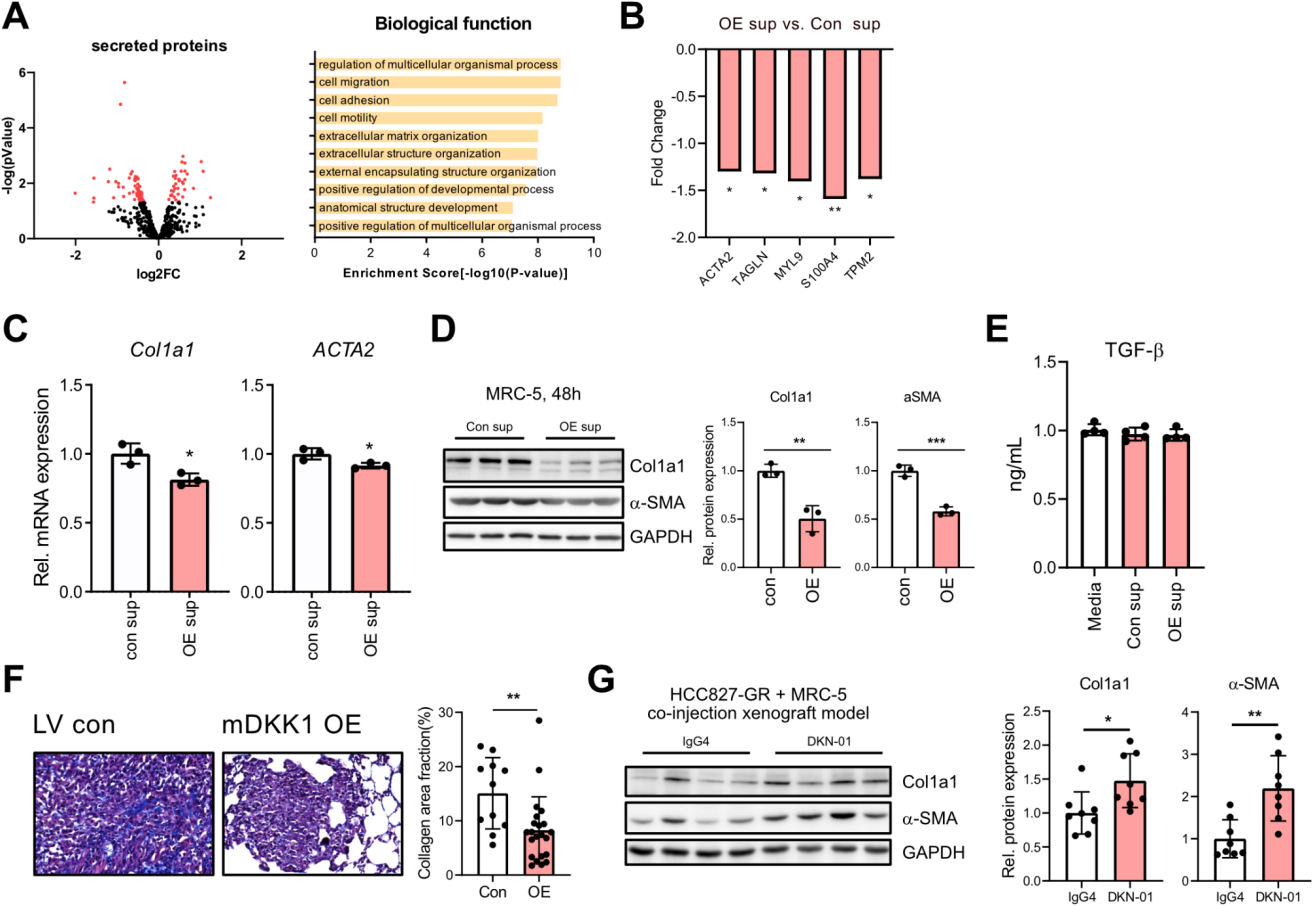
Decreases in myofibroblast characteristics of MRC-5 by DKK1. (**A**) Volcano plot for secretory genes using RNA-sequencing data from Fig. 6A. Red dots represent genes satisfying p-value < 0.05 (left). GO term analysis for secreted protein genes satisfying p-value < 0.05 (right). (**B**) Myofibroblast marker genes in MRC-5 treated with HCC827-DKK1-OE culture supernatants. The data were obtained using RNA-sequencing data from Fig. 6A. (**C**) *Col1a1*, *ACTA2* mRNA expression in MRC-5 treated with HCC827-DKK1-OE culture supernatants for 6 h. (**D**) Col1a1 and α-SMA protein expression in MRC-5 treated with HCC827-DKK1-OE culture supernatants for 48 h. (**E**) TGF-β concentration in culture media and supernatant obtained from HCC827-LV con and HCC827-DKK1 OE. (**F**) Masson’s trichrome staining using lung tissue obtained from Fig. 4F. Representative images (left) and collagen area fraction (%) analyzed by ImageJ software (right). (**G**) Protein levels of Col1a1 and a-SMA in tumor sample obtained from Fig. 5I. All statistical significance of the differences was determined by unpaired two-tailed Student t-test. ns, non-significant; *, *P* < 0.05; **, *P* < 0.01; ***, *P* < 0.001; ****, *P* < 0.0001

CAFs activated by cancer cells can differentiate into various phenotypes, including myofibroblast CAFs (myCAFs) involved in extracellular matrix (ECM) remodeling, inflammatory CAFs (iCAFs) with active inflammatory cytokine secretion, and antigen-presenting CAFs (apCAFs) [44]. Given that DKK1 promotes inflammatory cytokine secretion, thus activating iCAF characteristics, we investigated whether it also affects myCAF traits. Gene ontology analysis was performed among the MRC-5 secretory genes significantly altered by DKK1. This analysis revealed significant enrichment change in extracellular matrix formation-related ontologies (Fig. 7A). The expression of representative myofibroblast markers, including *ACTA2*, *TAGLN*, *MYL9*, *S100A4*, and *TPM2* was significantly reduced in the HCC827-DKK1 OE-treated group (Fig. 7B). Furthermore, both mRNA and protein levels of collagen and *ACTA2* of MRC-5 were significantly decreased following treatment with HCC827-DKK1 OE culture media (Fig. 7C and D). Because TGF-β is the most well-known factor that induces myofibroblast activation, we investigated whether the observed changes in MRC-5 were caused by TGF-β present in the cancer cell supernatant. However, neither HCC827-LV con nor HCC827-DKK1 OE produced TGF-β (Fig. 7E), indicating that the reduction of myofibroblast traits by DKK1 is not due to changes in TGF-β production by cancer cells.

To confirm this phenomenon in animal samples, Masson’s trichrome collagen staining was performed on 4T1-mDKK1 OE lung cancer tissue. The distribution of collagen was reduced in tumors composed of mDKK1-overexpressing cells (Fig. 7F). Conversely, cancer tissue with blocked DKK1 function, achieved by administering a DKK1-neutralizing antibody, exhibited increased collagen and α-SMA protein levels (Fig. 7G). Collectively, these results indicate that DKK1 reduces the myofibroblast properties of lung fibroblasts.

### Cancer cell-derived DKK1 activates lung fibroblast via the c-Jun N-terminal kinase (JNK)/c-JUN pathway

**Fig. 8:**
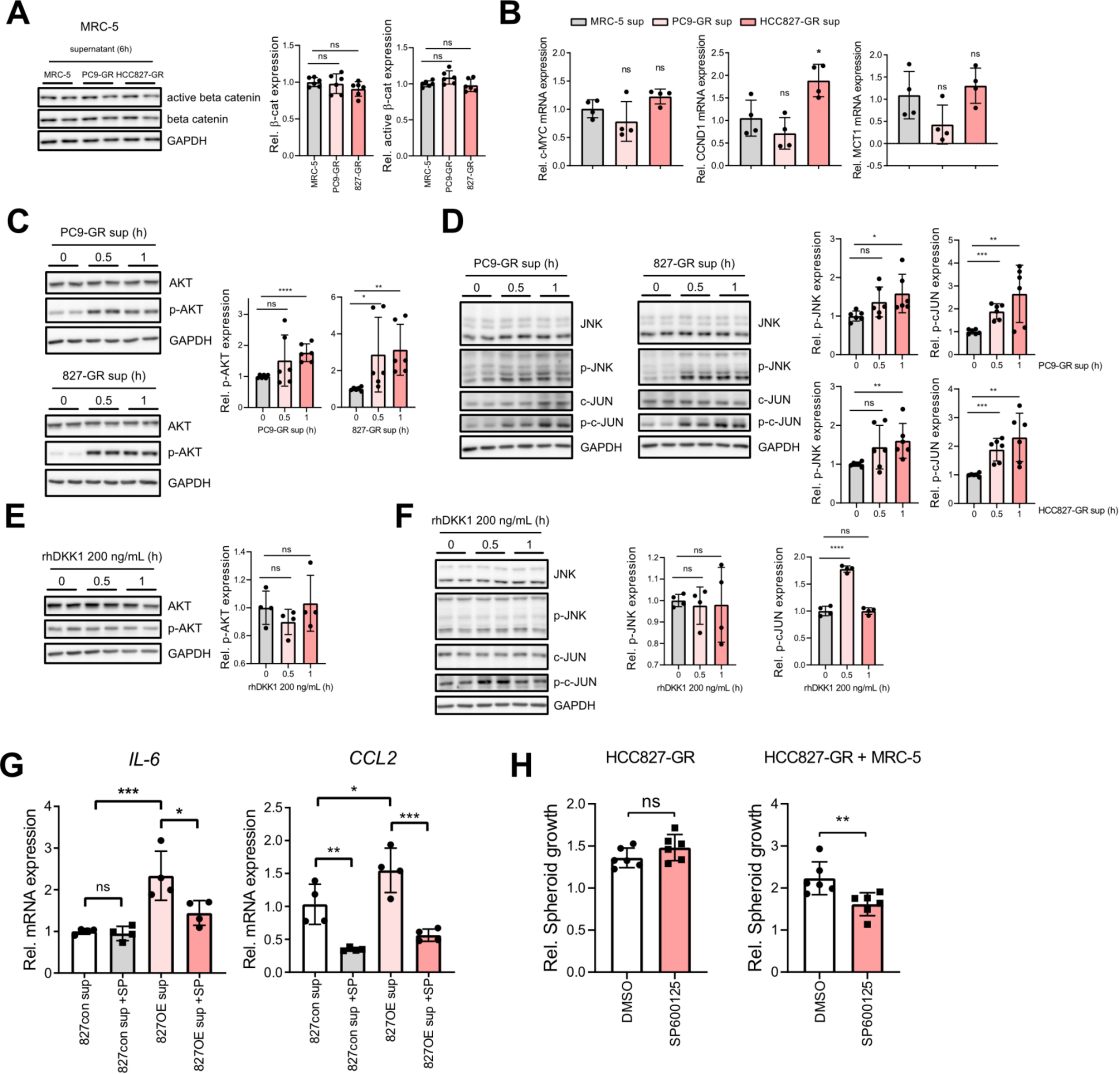
c-JUN pathway activation in fibroblast by DKK1. (**A** and **B**) Active β-catenin or total β-catenin protein expression(**A**) and β-catenin downstream genes expression (**B**) in MRC-5 treated with PC9-GR or HCC827-GR supernatant for 6 h. (**C** and **D**) Phosphorylation of AKT (**C**) and JNK and c-JUN (**D**) in MRC-5 treated with PC9-GR or HCC827-GR supernatant for 30 min–1 h. (**E** and **F**) Phosphorylation of AKT (**E**) and JNK and c-JUN (**F**) in MRC-5 treated with 200 ng/mL of recombinant DKK1 protein for 30 min–1 h. (**G**) Effects of SP600125 (10 µM) on *IL-6* and *CCL2* mRNA expression in MRC-5 treated with HCC827-LV con or HCC827-DKK1-OE culture supernatant for 6 h. (**H**) The volume of 3D spheroid containing HCC827-GR or HCC827-GR with MRC-5 in the presence of 10 µM SP600125. The data shows the relative spheroid volumes after 6 days. All statistical significance of the differences was determined by unpaired two-tailed Student t-test except (**G**). one-way ANOVA followed by Tukey post hoc test (**G**). ns, non-significant; *, *P* < 0.05; **, *P* < 0.01; ***, *P* < 0.001; ****, *P* < 0.0001

Next, we sought to elucidate the mechanism by which DKK1 affects fibroblasts. DKK1 is known as a negative regulator of the β-catenin dependent Wnt signaling pathway [40]. DKK1 binds to LRP5/6 in a competitive manner with Wnt, inhibiting β-catenin dependent Wnt signaling pathway [45]. Thus, we examined whether culture medium from GR cells could alter β-catenin protein levels or activity in MRC-5 cells. However, GR cell supernatant did not affect β-catenin protein levels or active β-catenin (Fig. 8A). Furthermore, mRNA levels of several β-catenin downstream target genes (*MYC*,* CCND1*,* MCT1*) showed no significant changes following GR cell supernatant treatment (Fig. 8B), excluding the hypothesis that DKK1 affects the β-catenin dependent Wnt pathway in MRC-5 cells.

DKK1 can also bind to CKAP4 and activate the downstream AKT pathway [18]. Unlike the β-catenin dependent Wnt pathway, GR cell culture medium induced AKT phosphorylation in MRC-5 cells (Fig. 8C). However, recombinant human DKK1 protein did not activate the AKT pathway in MRC-5 cells, indicating that the AKT pathway is not involved in DKK1-mediated fibroblast activation (Fig. 8E).

DKK1 has been reported to be involved in the β-catenin independent Wnt pathway. Frizzled (FZD) is another receptor for Wnt, leading to β-catenin independent signaling [46]. Since DKK1 blocks Wnt binding to LRP5/6, Wnt binding to FZD and its downstream signaling could be enhanced by DKK1 [19]. Indeed, several studies have shown that DKK1 activates the JNK/c-JUN pathway, which is downstream of FZD [47–49]. GR cell-culture medium effectively induced the phosphorylated form of JNK and c-JUN protein in MRC-5 cells (Fig. 8D). Recombinant human DKK1 also elevated phosphorylated c-JUN levels (Fig. 8F). Interestingly, the c-JUN pathway is an upstream regulator of inflammatory cytokine expression, including *IL-6* and *CCL2* [50]. We found that mRNA expression levels of *IL-6* and *CCL2* by HCC827-DKK1 OE supernatant were reduced by SP600125, a JNK inhibitor (Fig. 8G). Furthermore, SP600125 effectively reduced spheroid growth only when the spheroid was composed of GR cells with fibroblasts (Fig. 8H). Collectively, these results suggest that the c-JUN pathway in lung fibroblasts can be activated by DKK1, contributing to the activation of inflammatory fibroblast traits and cancer cell-fibroblast interaction.

### The roles of DKK1 in DKK1 high expressing tumors beyond gefitinib-resistant tumors

Next, we examined whether the DKK1 has a similar intratumoral role in high DKK1-expressing tumors beyond GR tumors. Using Depamp analysis data [33–37], we compared DKK1 expression across various cancer cell lines. A prostate cancer cell line, PC3, and a breast cancer cell line, MDA-MB-231 exhibited high levels of DKK1 expression (supplementary Figure S5A), confirmed by high DKK1 protein levels in their culture media (supplementary Figure S5B). Using these cell lines, the spheroid growth rates were compared. Co-culture with MRC-5 enhanced the spheroid growth in both cell lines (supplementary Figure S5C). In addition, culture media from PC3 and MDA-MB-231 elevated *IL-6* and *CCL2* mRNA expression and reduced collagen protein expression in MRC-5 (supplementary Figure S5D and S5E), indicating that high DKK1 expression alters fibroblast characteristics. Furthermore, culture medium from both cell lines activated the c-JUN pathway in MRC-5 cells, similar to GR cells (supplementary Figure S5F). Taken together, these results suggest that DKK1 contributes to fibroblast activation and cancer cell-fibroblast interaction in high DKK1-expressing tumors, highlightning DKK1 as a potential therapeutic target across various cancer types, not just in gefitinib-resistant NSCLC.

## Discussion

**Fig. 9:**
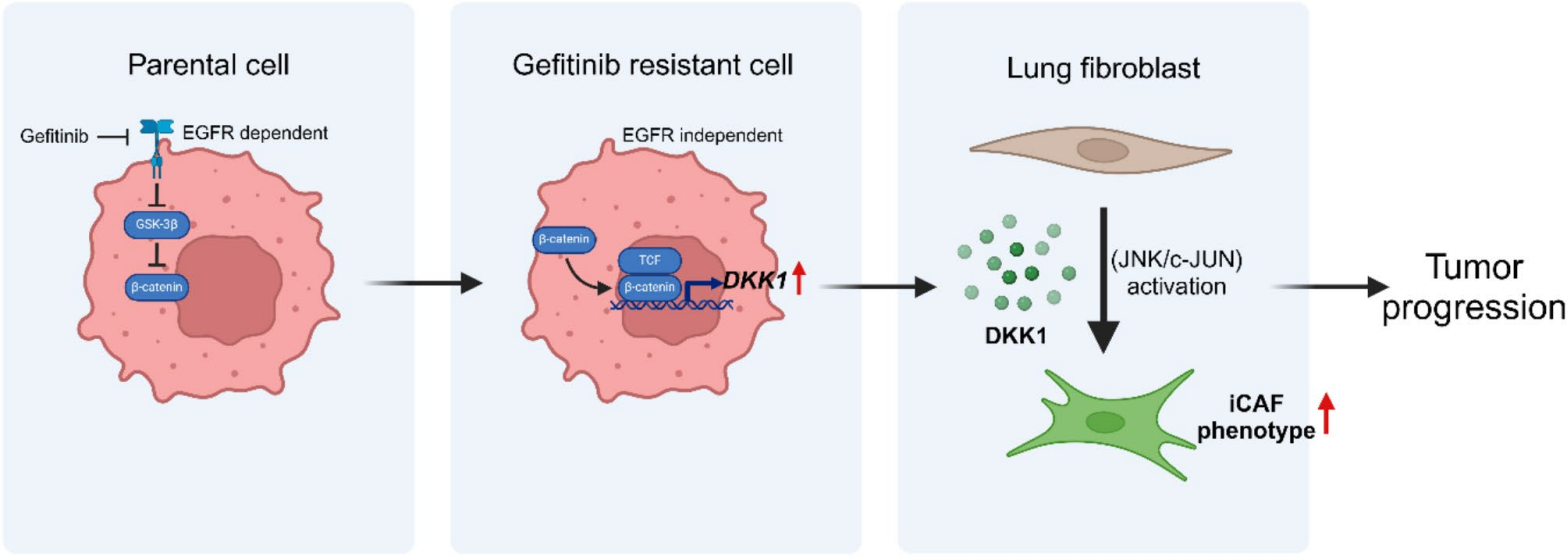
Graphical summary for this study

DKK1 is a secretory protein that functions as a mediator of intercellular interactions. Initially reported as an inhibitor of the oncogenic Wnt signaling pathway, DKK1 was originally considered a tumor suppressor gene [51]. However, subsequent studies have shown that DKK1 also contributes to tumor progression [19]. For example, Kasoha et al. have reported that DKK1 protein expression level is highly correlated with bone metastasis in breast cancer patients [52]. In addition, several reports have shown that DKK1 is correlated with the progression of hepatocellular carcinoma by inducing angiogenesis [53] or metastasis [54]. DKK1 has also been implicated in drug resistance [55, 56]. For example, the downstream pathways of DKK1 can modulate cancer cells to obtain resistance to drugs [57–59], and immune modulation by DKK1 can support resistance to immunotherapy [55, 60]. Here, we found that DKK1 expression and secretion were significantly increased in gefitinib-resistant NSCLCs, highlighting the role of DKK1 in the specific tumor microenvironment. Cancer cell-derived DKK1 mediates the interaction between cancer cells and fibroblasts, resulting in changes in fibroblast characteristics and subsequent tumor progression (Fig. 9). Based on these findings, we propose DKK1 as a therapeutic target for gefitinib-resistant NSCLCs.

CAFs can be classified to several subpopulations including iCAFs, myCAFs, and apCAFs [44]. Among them, iCAFs can support the tumor progression by secreting various cytokines [61]. For example, iCAF-derived IL-6 induces epithelial-mesenchymal transition (EMT) [62] and cell proliferation [63] of cancer cells. Besides, hepatocyte growth factor (HGF) secreted from iCAF promotes tumor growth by activating its receptor, Met on cancer cell [64]. Here, we showed that lung fibroblast activated by DKK1 actively produced IL-6 and CCL2, which are representative cytokines identified as the iCAF markers, which raise a notion that iCAFs are activated and promote tumor progression in high DKK1-expressing tumors.

The DKK1 promoter contains several TCF binding elements (TBEs) to which the β-catenin/TCF complex can bind [40]. Indeed, it has been reported that *DKK1* transcription is regulated by β-catenin [65, 66]. Similarly, DKK1 expression was regulated by β-catenin in the two gefitinib-resistant cell lines used in this study. Therefore, the activity of β-catenin seems to be crucial in determining the expression level of DKK1. The activity of β-catenin can be regulated by various factors. For example, DKK1 inhibition of Wnt signaling promotes β-catenin proteolysis and prevents its nuclear translocation [17]. Besides, binding to proteins such as LEF1 or MUC-1 facilitates β-catenin’s nuclear translocation [67, 68], while binding to Axin or other partners can sequester β-catenin away from the nucleus and hold it on other locations such as cell membrane [69, 70]. Therefore, further studies are needed to elucidate the precise mechanisms regulating β-catenin activity in gefitinib-resistant cells to understand the increase in DKK1.

Although the mechanism regulating β-catenin activity in resistant cells is unclear, it is believed that DKK1 expression could be increased along the various type of gefitinib-resistant cells. According to the previous reports, EGFR-TKI resistance mechanisms are commonly related to β-catenin activation. For example, β-catenin pathway is activated in cells carrying T790M mutation [71]. Similarly, MET amplification, another mechanism to EGFR-TKI, has also been shown to activate β-catenin pathway [72]. Furthermore, β-catenin pathway is linked to the EMT pathway, suggesting that β-catenin activation occurs in EGFR-TKI resistant cells undergoing EMT [73]. Thus, β-catenin activation appears to be a common feature among several resistance mechanisms, which may explain the elevated DKK1 levels observed in various types of EGFR-TKI resistant cells.

Previous studies have reported that DKK1 affects immune cells within the tumor. For example, cancer cell-derived DKK1 can inhibit the tumor infiltration of macrophages and neutrophils resulting in the immune suppressive TME [22]. Furthermore, it has been reported that DKK1 enhances the immune suppressive function of myeloid-derived suppressor cells (MDSC) [74] and suppresses the activity of T lymphocytes [75]. Considering that DKK1 enhances the inflammatory traits of fibroblasts, it is possible that cytokines secreted from activated fibroblasts could impact immune cells. This suggests that DKK1 influences immune cells indirectly through fibroblasts in addition to its direct effects on immune cells, indicating the possible role of DKK1 in the overall TME. In this study, we did not consider the immune system’s effect since the co-culture of cancer cells and fibroblasts without immune cells promoted spheroid growth in vitro. Moreover, a DKK1 neutralizing antibody demonstrated anti-tumor effects in immune-deficient mice. However, as previously described, DKK1 acts as a mediator for comprehensive interactions within the TME. Therefore, further research incorporating immune cells using allograft or humanized mouse models would be necessary to fully establish DKK1’s roles in cancer patients.

In the final part of this study, we confirmed that DKK1 has similar intratumoral roles in other high DKK1-expressing tumors besides gefitinib-resistant tumors. This finding suggests that DKK1 could be a potential anticancer target across various cancer types with high DKK1 expression. Given that DKK1 is a secretory protein detectable in blood [76], serum DKK1 levels could serve as a useful indicator of the TME and the efficacy of DKK1-targeting agents.

## Conclusion

In conclusion, we found that DKK1 expression was elevated in gefitinib-resistant NSCLC cells via the increased transcriptional activity of β-catenin. Cancer cell-derived DKK1 contributes to tumor progression by mediating interactions between cancer cells and fibroblasts within the tumor, rather than directly affecting the cancer cells. This study is the first to report the effects of DKK1 on fibroblasts in tumors, showing that DKK1 activates c-JUN signaling in fibroblasts and enhances their inflammatory traits. Thus, we propose DKK1 as a novel anticancer target for treating gefitinib-resistant NSCLC.

## Electronic Supplementary Material

Below is the link to the electronic supplementary material.

Supplementary Material 1

## Data Availability

RNA sequencing data been deposited in NCBI’s Gene Expression Omnibus(GSE278343). GSE121634 data set was obtained from NCBI GEO database and analyzed. The expression profile of DKK1 protein in lung cancer patient was obtained from oncoDB(https://oncodb.org/). Overall survival rate of DKK1 low- or high-patients were obtained from Kaplan-Meier Plotter(https://kmplot.com/analysis/).. DKK1 protein expression and gene dependency data were analyzed using DepMap(https://depmap.org/portal/).

## Abbreviations

apCAF: antigen presenting CAF
CAF: Cancer-Associated Fibroblast
CCL2: C-C motif chemokine ligand 2
CKAP4: Cytoskeleton-Associated Protein 4
DKK1: Dickkopf-1
DMEM: Dulbecco’s Modified Eagles Medium
EGFR: Epidermal Growth Factor Receptor
GEO: Gene Expression Omnibus
GR: Gefitinib-Resistant
GSEA: Gene Set Enrichment Analysis
H&E: Hematoxylin and Eosin
iCAF: inflammatory CAF
IL-6: Interleukin 6
LRP5/6: Lipoprotein receptor-related protein 5/6
myCAF: myofibroblastic CAF
MDSC: Myeloid-Derived Suppressor Cell
NSCLC: Non-Small Cell Lung Cancer
OE: Over-Expression
TBE: TCF Binding Element
TKIs: Tyrosine Kinase Inhibitors
TME: Tumor Microenvironment
