# Supplementary material for: Dickkopf-1 promotes tumor progression of gefitinib- resistant non-small cell lung cancer through cancer cell-fibroblast interactions
Source: Exp Hematol Oncol. 2025 Mar 1;14:24. doi: 10.1186/s40164-025-00616-9 (PMC11871833; doi:10.1186/s40164-025-00616-9)
Supplement: Supplementary file 1 — Supplementary Material 1 [file 40164_2025_616_MOESM1_ESM.docx]

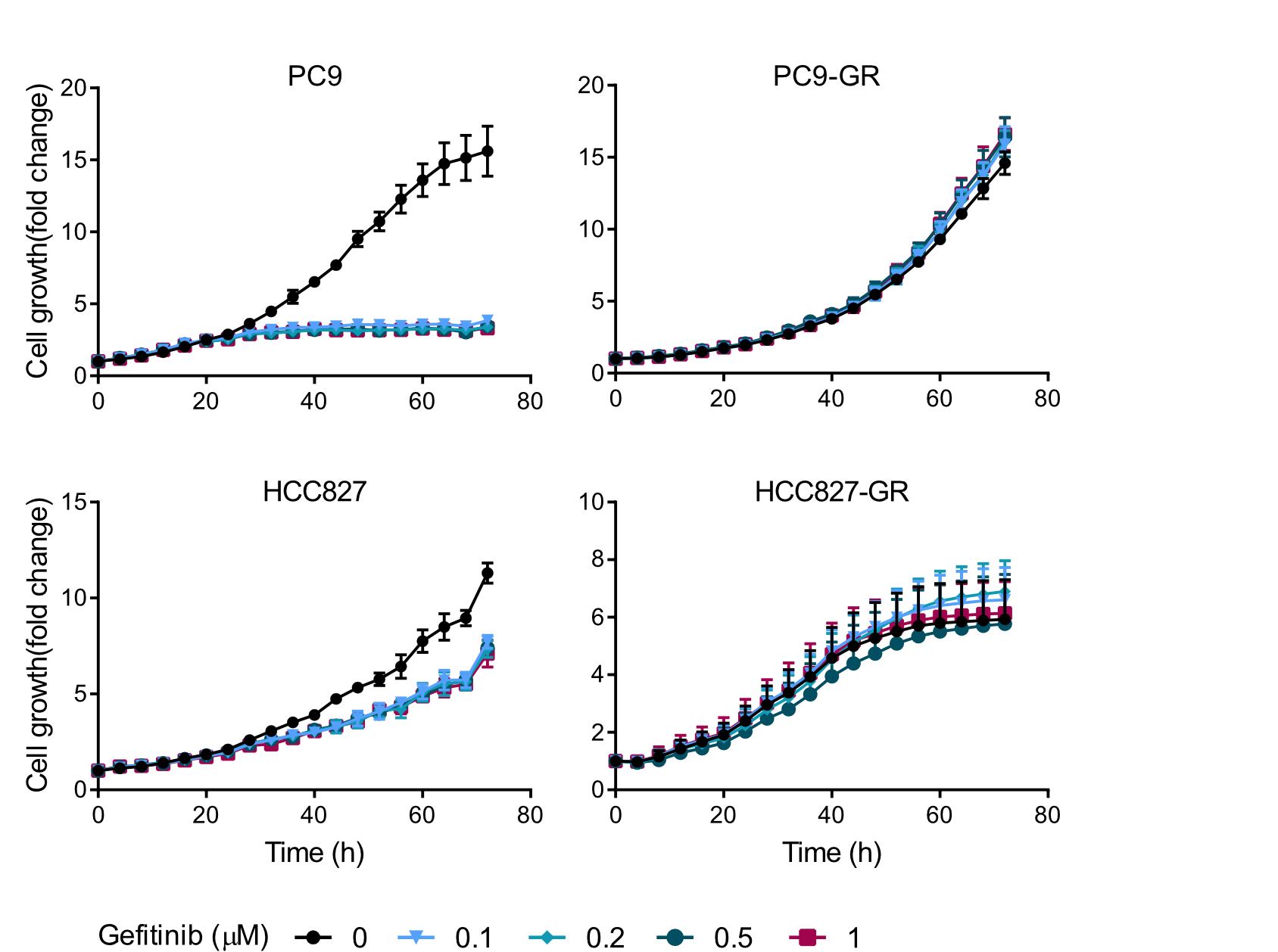


**Supplementary Figure 1. Inhibition of cell proliferation by gefitinib in parental and gefitinib-resistant NSCLC cell lines (PC9-GR and HCC827-GR).** Parental and gefitinib-resistant NSCLC cells were treated with 0, 0.1, 0.2, 0.5, and 1 μM gefitinib, and cell proliferation was monitored by IncuCyte S3.


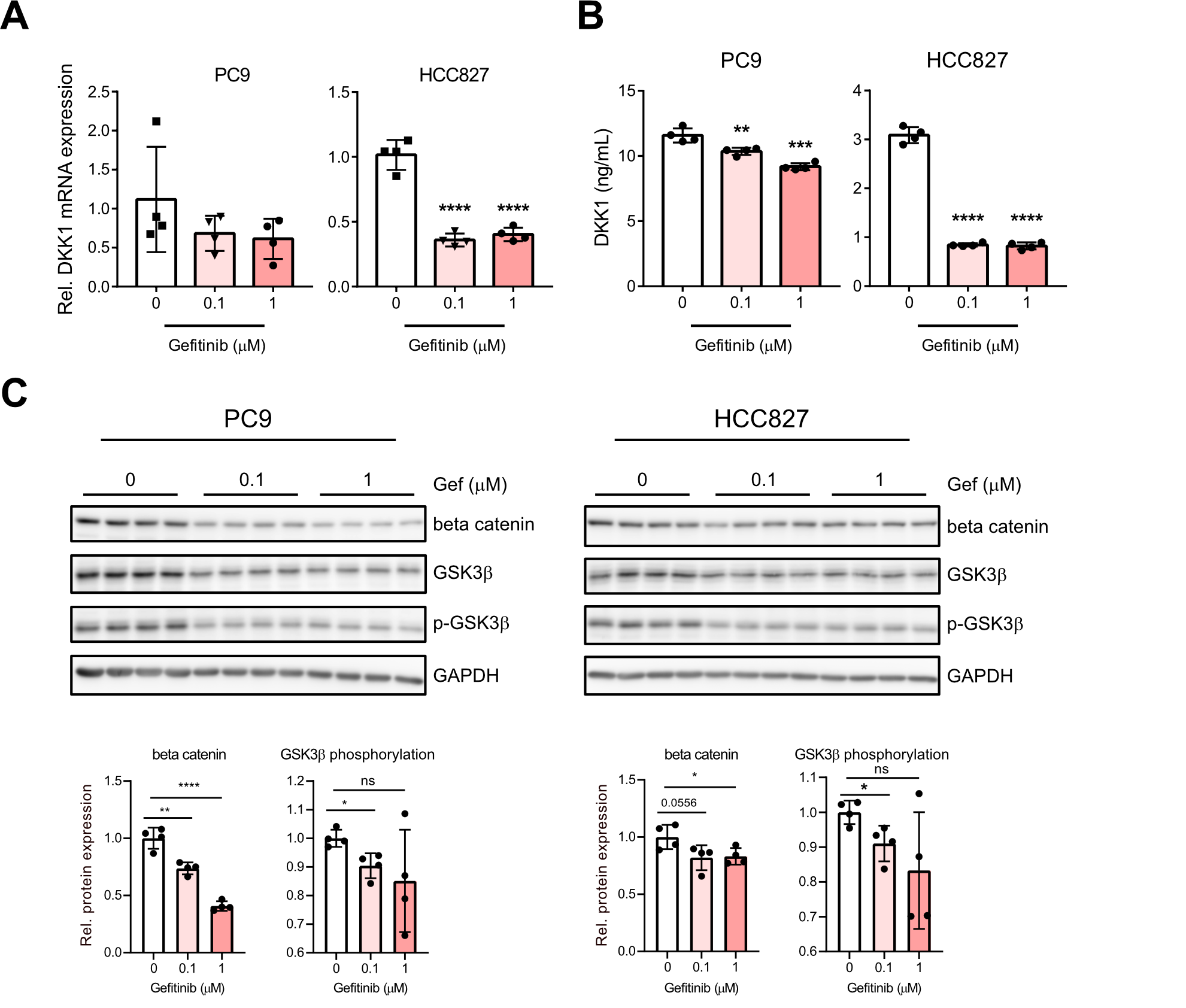


**Supplementary Figure 2. Gefitinib treatment reduces DKK1 expression in NSCLC cell lines**

**(A and B)** The expression levels of *DKK1* mRNA(**A**) and protein (**B**) in PC9 and HCC827 treated with gefitinib for 24 h. **(C)** Changes in GSK-3β phosphorylation and β-catenin protein levels by gefitinib treatment. Total cell lysates were obtained 48 h after gefitinib exposure. All statistical significance of the differences was determined by unpaired two-tailed Student t-test. ns, non-significant; *, P < 0.05; **, P < 0.01; ***, P < 0.001; ****, P < 0.0001.


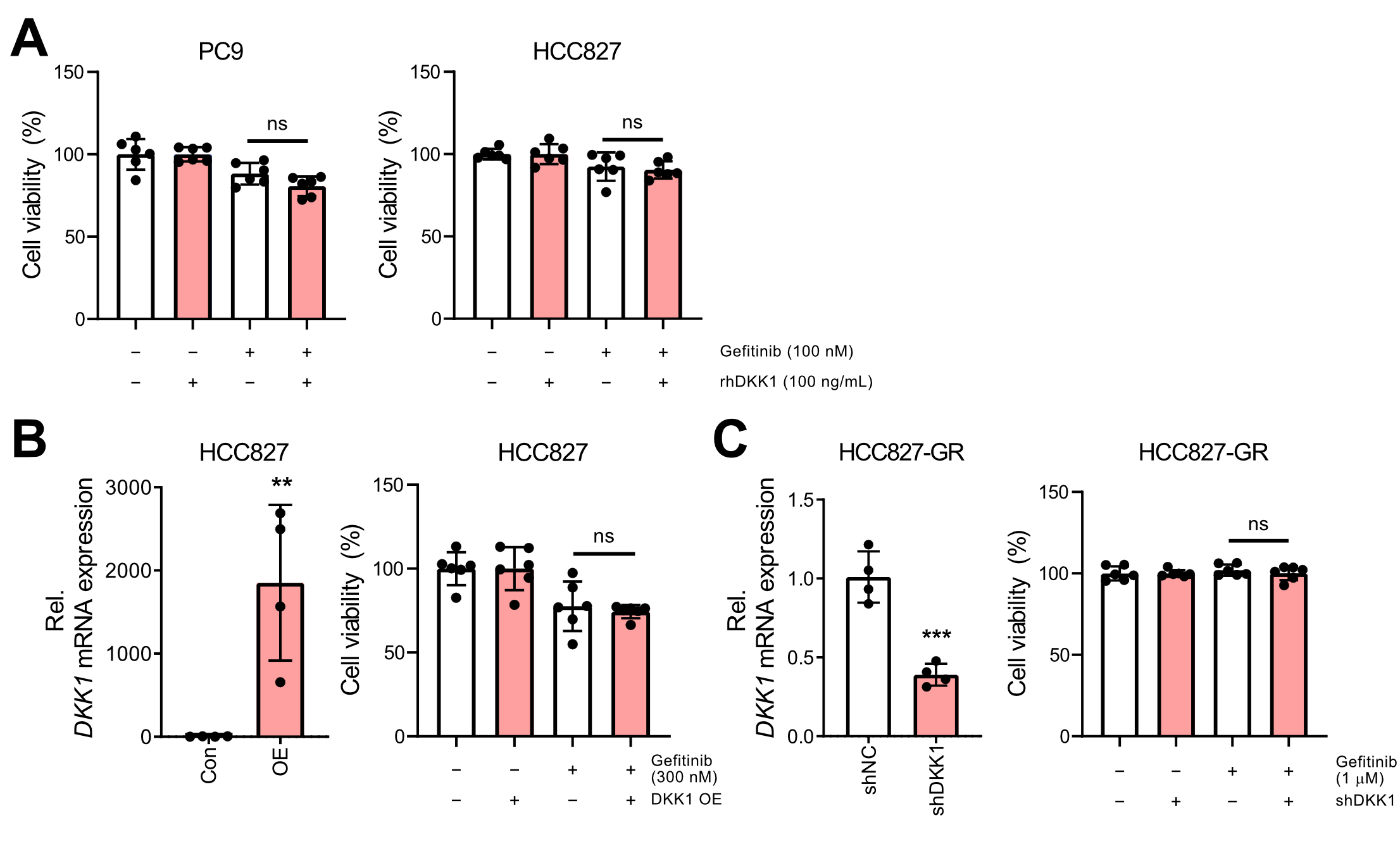


**Supplementary Figure 3. Sensitivity to gefitinib is not affected by DKK1 expression**

**(A)** Cell viability of NSCLC cells. PC9 and HCC827 were incubated with or without 100 ng/mL of rhDKK1 in the presence or absence of 100 nM gefitinib for 24 h. **(B)** *DKK1* mRNA expression in HCC827-LV con and HCC827-DKK1-OE(left). Cell viability of HCC827-LV con and HCC827-DKK1-OE 48 h after 300 nM gefitinib exposure(right). **(C)** *DKK1* mRNA expression in HCC827-shNC con and HCC827-shDKK1(left). Cell viability of HCC827-GR-shNC and HCC827-GR-shDKK1 48 h after 1 μM gefitinib exposure(right). All statistical significance of the differences was determined by unpaired two-tailed Student t-test. ns, non-significant; *, P < 0.05; **, P < 0.01; ***, P < 0.001; ****, P < 0.0001.


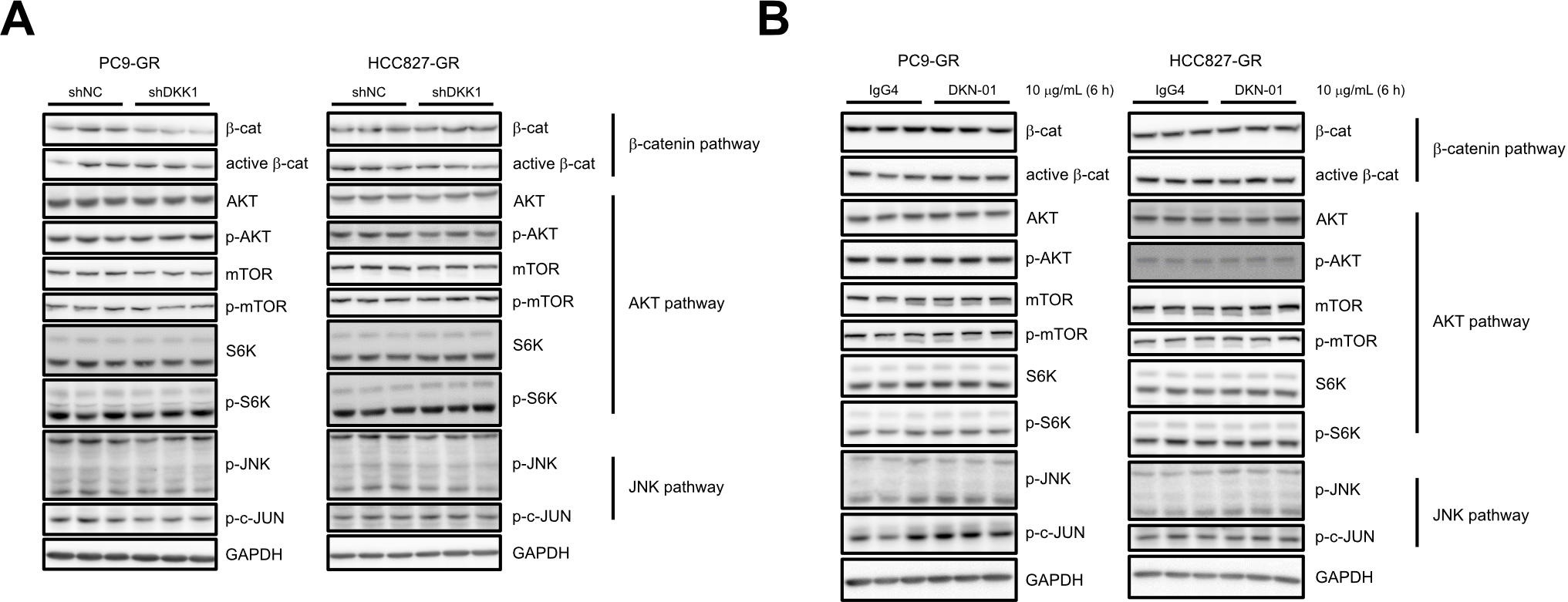


**Supplementary Figure 4. No changes in DKK1 downstream pathways by DKK1 blockade in gefitinib- resistant cells**

**(A and B)** Proteins expression levels of β-catenin, AKT and JNK/c-JUN pathway in gefitinib- resistant cells (PC9-GR d HCC827-GR) after knockdown of DKK1(**A**) or treatment of DKN-01(**B**).


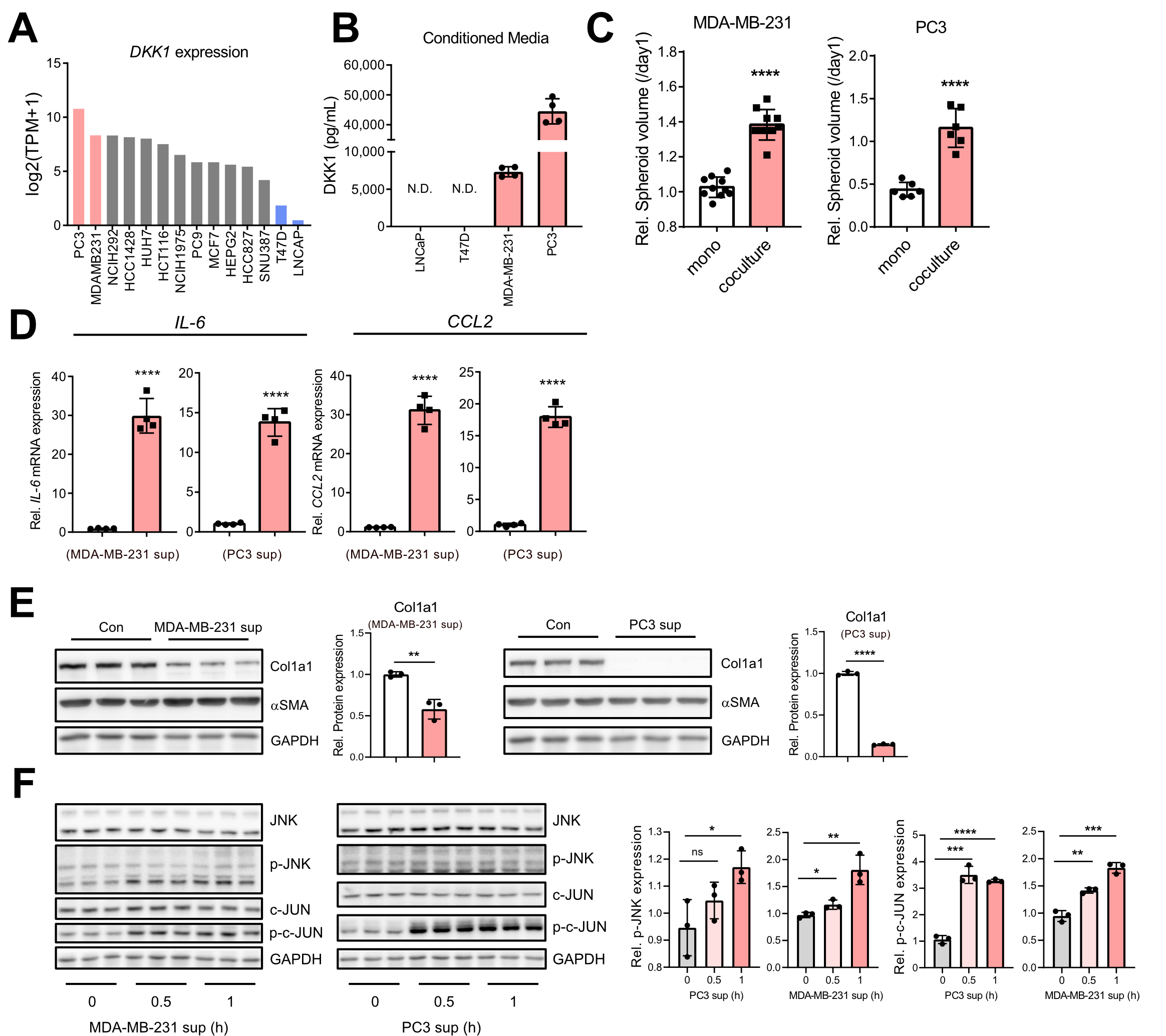


**Supplementary Figure 5. DKK1 high expressing cancer cells can also activate fibroblast**

**(A)** *DKK1* mRNA expression levels of the variety of cancer cell lines. Data was collected from Depmap. **(B)** DKK1 protein levels in culture media of LNCaP, T47D, MDA-MB-231 and PC3. **(C)** 3D spheroid growth rates of cancer cell (MDA-MB-231 or PC3) only or cancer cell cocultured with MRC-5. **(D)** The elevation of *IL-6* and *CCL2* mRNA levels in MRC-5 treated with MDA-MB-231 and PC3 culture supernatants for 6 h. **(E)** Col1a1 and α-SMA protein expression in MRC-5 treated with MDA-MB-231 and PC3 culture supernatants for 48 h. **(F)** Increased phosphorylation of JNK and c-JUN in MRC-5 treated with MDA-MB-231 and PC3 culture supernatants for 30 min or 1 h. All statistical significance of the differences was determined by unpaired two-tailed Student t-test. ns, non-significant; *, P < 0.05; **, P < 0.01; ***, P < 0.001; ****, P < 0.0001.

**
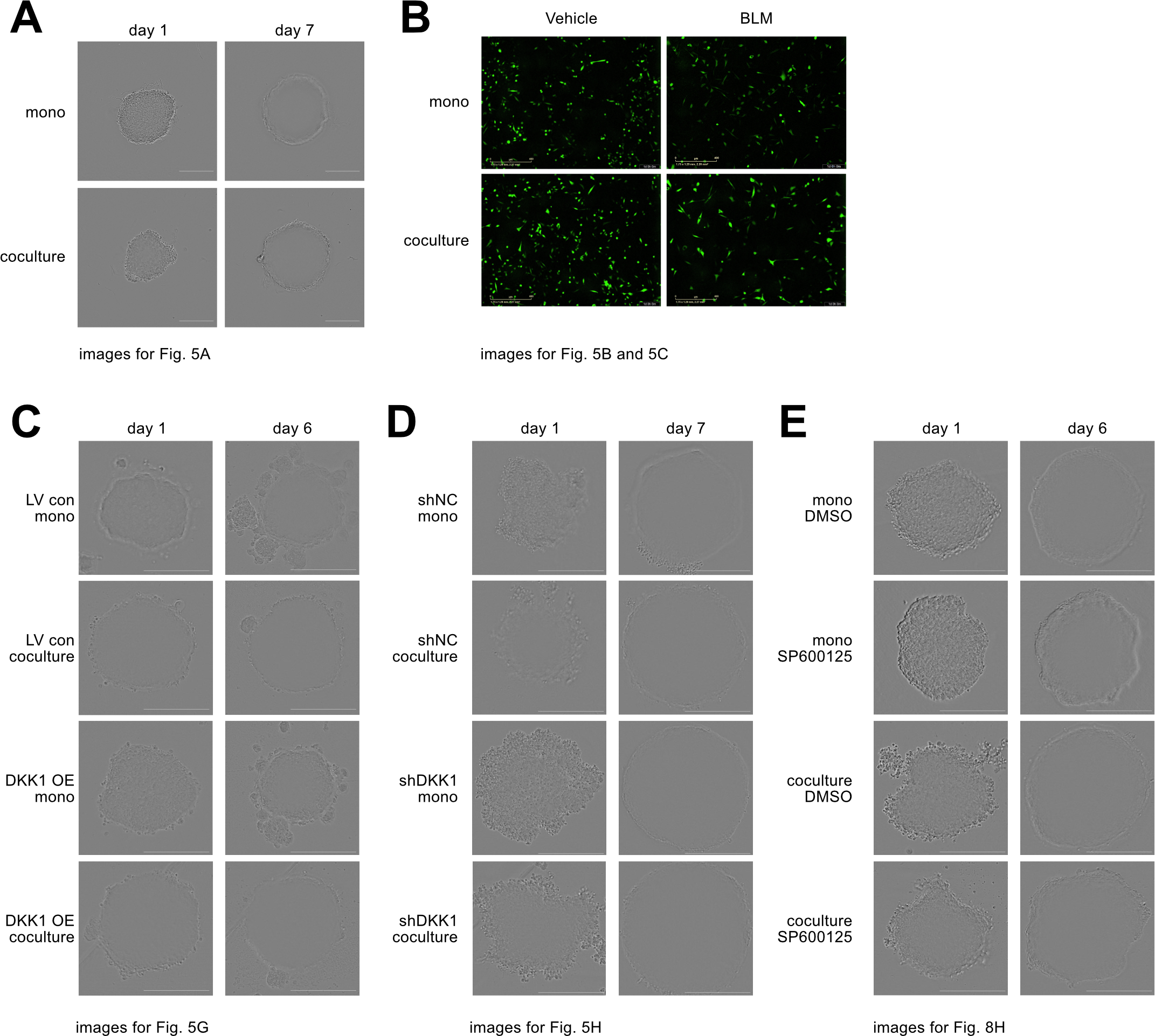
**

**Supplementary Figure 6. Images for spheroid assay and GFP measurement results**

**(A-E)** The images obtained by IncuCyte S3 for main Figure 5A(**A**), 5B and 5C(**B**), 5G(**C**), 5H(**D**), and 8H(**E**). Scale bars for spheroid images represent 400 μm.
